# Watching right and wrong nucleotide insertion captures hidden polymerase fidelity checkpoints

**DOI:** 10.1038/s41467-022-30141-w

**Published:** 2022-06-09

**Authors:** Joonas A. Jamsen, David D. Shock, Samuel H. Wilson

**Affiliations:** Genome Integrity and Structural Biology Laboratory, National Institute of Environmental Health Sciences, National Institutes of Health, Research Triangle Park, NC 27709 USA

**Keywords:** X-ray crystallography, Cancer, Enzyme mechanisms

## Abstract

Efficient and accurate DNA synthesis is enabled by DNA polymerase fidelity checkpoints that promote insertion of the right instead of wrong nucleotide. Erroneous X-family polymerase (pol) λ nucleotide insertion leads to genomic instability in double strand break and base-excision repair. Here, time-lapse crystallography captures intermediate catalytic states of pol λ undergoing right and wrong natural nucleotide insertion. The revealed nucleotide sensing mechanism responds to base pair geometry through active site deformation to regulate global polymerase-substrate complex alignment in support of distinct optimal (right) or suboptimal (wrong) reaction pathways. An induced fit during wrong but not right insertion, and associated metal, substrate, side chain and pyrophosphate reaction dynamics modulated nucleotide insertion. A third active site metal hastened right but not wrong insertion and was not essential for DNA synthesis. The previously hidden fidelity checkpoints uncovered reveal fundamental strategies of polymerase DNA repair synthesis in genomic instability.

## Abstract (web summary)

DNA polymerase (pol) λ performs DNA synthesis in base excision and double strand break repair. How pol λ accomplishes nucleotide insertion that can lead to mutagenesis and genomic instability was unclear. Here the authors employ time-lapse crystallography to reveal hidden polymerase checkpoints that enable right and wrong natural nucleotide insertion by pol λ.

## Introduction

Unrepaired DNA double-strand breaks (DSBs) are highly cytotoxic and lead to genomic instability. X-family DNA repair polymerase^1^ (pol) λ performs gap-filling DNA synthesis during base-excision repair (BER) and DSB repair^2–4^. Repair synthesis by pol λ is known to introduce mismatches, short deletions, and insertions at the repaired break site^5–7^. Erroneous pol λ DNA synthesis is a crucial factor towards understanding genome instability in cancer, aging, disease and evolution^8^.

DNA polymerase closing is a well-known fidelity checkpoint that aligns the primer terminus and positions active site catalytic residues for nucleotide insertion. X-family DNA polymerase β, for example, undergoes subdomain repositioning upon nucleotide binding to the pol-DNA binary complex to create the pol-DNA-dNTP ternary complex^9–11^. Inefficient subdomain closing and/or active site misalignment post-closing enables inefficient misinsertion of a wrong nucleotide. Pol λ has been reported to lack conformational transitions during its catalytic cycle. A shift in the DNA substrate instead enables nucleotide insertion^12^.

Polymerase DNA synthesis has been extensively studied using chemical analogues of natural nucleotides to trap catalytically informative conformational states of polymerases^12–18^. Full appreciation of the molecular basis of polymerase DNA synthesis has been constrained by the lack of structural information on natural nucleotide insertion. Here, time-lapse crystallography with right (matched) and wrong (mismatched) natural nucleotides captures snapshots of pol λ undergoing DNA synthesis. The intermediate catalytic states uncovered reveal hidden fidelity checkpoints that enable DNA polymerase discrimination between right and wrong nucleotides.

## Results

Time-lapse crystallography yields snapshots of catalytic events as a DNA polymerase transitions through the reaction cycle of nucleotide insertion from nucleotide binding to product formation and PP_i_ release^19–26^. The approach routinely reveals transient intermediate states and conformational transitions during DNA synthesis. Here, we applied time-lapse crystallography to observe right and wrong nucleotide insertion by DNA polymerase λ.

### Matched ground state

Time-lapse crystallography of X-family pols β^19,20,25–27^ and μ^22^, as well as Y-family polymerase η^21,24^, involved growing polymerase–DNA binary complex crystals, and soaking these crystals in the presence of dNTP and Ca^2+^ to generate pol–DNA–dNTP ternary ground state (Ca^2+^-GS) complex crystals^19–27^. The addition of Ca^2+^ allows dNTP binding but does not support catalysis of nucleotide insertion in crystallo. We grew crystals of the catalytic domain of wild-type pol λ^7,12,17,18^ with a single-nucleotide gapped DNA with template deoxyadenosine (A_t_) in the gap. We then soaked these crystals in a cryo-solution containing thymidine 5´-triphosphate (TTP) and Ca^2+^. These crystals diffracted to 2.3 Å and were unsuitable for the routine soaking required in the time-lapse crystallography approach. Ternary complex crystals of the catalytic domain of pol λ with a truncated loop 1 (pol λ DL) grown in the presence of Ca^2+^ diffracted to higher resolution and were employed in subsequent soaks. Pol λ DL displays identical insertion kinetics and active site structure as the catalytic domain of wild-type pol λ^14,28^.

**Fig. 1:**
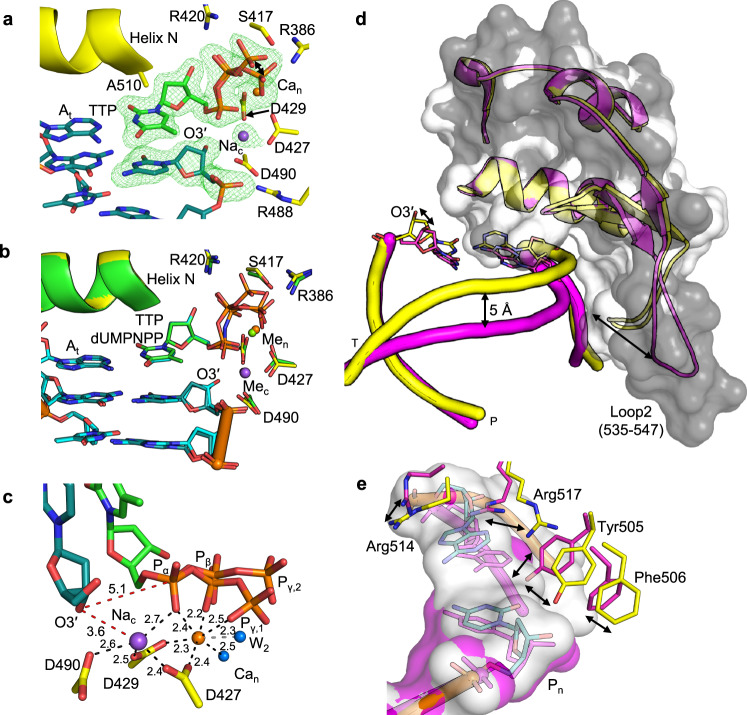
Matched ground state ternary complex. **a** Active site of the matched (TTP:A_t_) Ca^2+^-ground state (GS) ternary complex. Incoming thymidine 5′-triphosphate (TTP) opposite template deoxyadenosine (A_t_) is displayed in green stick representation, DNA in cyan, active site residues in yellow. Ca^2+^ bound to the nucleotide metal site (Ca_n_) is shown as an orange sphere, catalytic Na^+^ (Na_c_) is a purple sphere. Dynamics in the position of P_γ_ (of TTP) is indicated with a black double-headed arrow. Simulated annealing omit (F_o_-F_c_) density (green mesh) is contoured at 3 σ, carve radius 2.0 Å. **b** Structural overlay of the matched Ca^2+^-GS ternary complex and a pre-catalytic ternary complex (PDB id 2PFO^18^, protein green, DNA light cyan) bound to a non-hydrolyzable nucleotide (dUMPNPP, light cyan). Mg^2+^ is shown as a green sphere. **c** Active site metal coordination (Å, black dashes) and key distances (Å, red dashes) in the Ca^2+^-GS complex. The view shown is a ~90° rotation of that in panel (**a**). Distance of O3′ from both P_α_ and Na_c_ is shown with red dashes. Coordination of Ca_n_ with an alternate water (W_2_), when P_γ,2_ interacts with Arg420 (see Supplementary Fig. 1a), is shown with a grey dashed line. **d** Template strand shift in the matched Ca^2+^-GS ternary complex (yellow cartoon). Overlay with the structure of a pol λ-DNA binary complex (magenta cartoon, PDB id 1XSL^12^) is shown. A shift in loop 2 (Ala535 to Pro547) to contact the downstream template strand is observed upon TTP binding. Differences in atomic positions are shown with double-headed arrows. Atomic volume is shown in light and dark gray surface representation for the Ca^2+^-GS and binary complex, respectively. **e** Shifts in “steric gate” (Tyr505 and Phe506) and active site arginine residues (Arg514 and Arg517) upon ternary complex formation. Color schemes are as described above. Atomic volume is displayed as a light gray surface for the GS complex and in magenta for the binary complex. The template strand shift and associated changes in active site residues are shown with double-headed arrows.

Figure 1a shows the active site of the pre-catalytic Ca^2+^-GS ternary complex determined at 1.98 Å resolution with bound TTP opposite A_t_ (Supplementary Table 1). This Ca^2+^-GS complex is similar to previously determined pre-catalytic structures with a non-hydrolyzable incoming nucleotide analogue (Fig. 1b; PDB id 2PFO^18^). The lack of simulated annealing omit (F_o_-F_c_) density between the primer terminus O3´ and P_α_ of TTP indicates the absence of incorporation (Fig. 1a). The active site is in a catalytically incompatible state, as suggested by the C1´-exo conformation of the primer terminal deoxyribose sugar, and O3´ is positioned ~5 Å away from P_α_ (Fig. 1c). This distance is too far to allow nucleophilic attack at P_α_ and phosphodiester bond formation. As illustrated in Fig. 1c, while the nucleotide metal site is occupied by Ca^2+^ (Ca_n_), the tetrahedral coordination geometry, coordination distances, electron density, and lack of coordination with the primer O3´ (~3.6 Å) suggest that the catalytic metal site is occupied by Na^+^ (Na_c_). Triphosphate stabilization is achieved by interactions with the nucleotide metal, as well as with the side chains of Arg386, Ser417 and Arg420 (Fig. 1a). The elongated F_o_-F_c_ omit density surrounding P_γ_ suggests alternate coordination geometries with Ca_n_ and Arg386 (Fig. 1a–c, Supplementary Fig. 1a).

An overlay of the Ca^2+^-bound ternary complex with a binary complex structure (PDB id 1XSL)^12^ illustrates the robust ~5 Å template strand shift observed upon nucleotide binding that accompanies ternary complex formation (Fig. 1d, Supplementary Fig. 1b). A concurrent shift of a loop (loop2, Ala535 to Pro547) in the C-terminal polymerase subdomain toward the template strand is observed upon nucleotide binding to the binary DNA complex (Fig. 1d). Binding of TTP was, among other differences, accompanied by alterations of active site side chain conformations of residues in α-helix N (Arg514 and Arg517) and the catalytic subdomain (Tyr505 and Phe506) (Fig. 1e).

### Matched reaction state

**Fig. 2:**
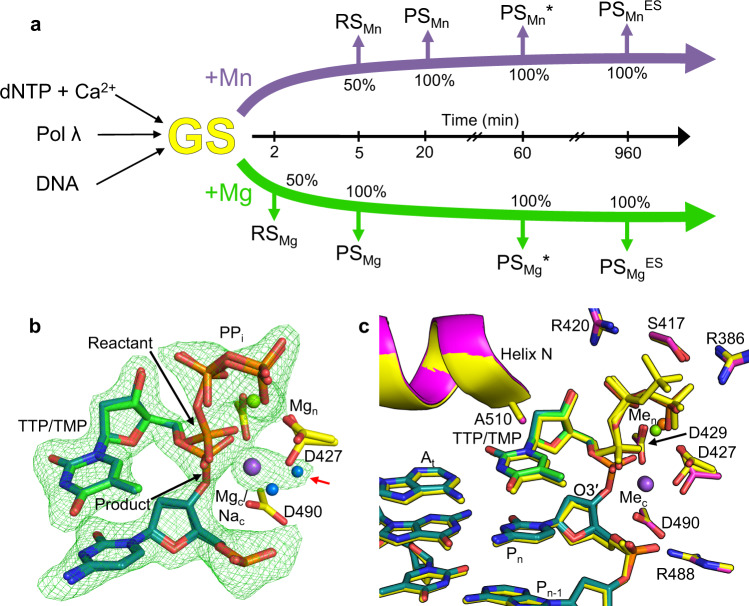
Time-lapse crystallography and the matched Mg^2+^-reaction state ternary complex. **a** Time-lapse x-ray crystallography protocol. Ground state ternary complex crystals (GS, yellow) were grown by mixing pol λ, gapped DNA substrate, nucleotide (dNTP), CaCl_2_ and precipitants. Ground state (GS) crystals were then soaked in a cryo-solution containing 50 mM Mg^2+^ (green arrow) or 50 mM Mn^2+^ (purple arrow) to initiate the reaction in the crystal. Crystals were frozen after increasing incubation times (central black arrow) and the structures of the intermediate complexes at increasing degrees of product formation (% incorporation) were determined. **b** Matched Mg^2+^-reaction state (RS_Mg_) structure after a 2 min soak. Partial bond breakage in substrate (P_α_–P_β_ of TTP) and bond formation in product (primer O3´–P_α_ of TTP) is visible, with inversion about P_α_ and concurrent PP_i_ formation. Rotation of Asp427 and presence of Mg^2+^ in the nucleotide metal site (Mg_n_) and Mg^2+^/Na^+^ (Mg_c_/Na_c_) in the catalytic metal site, is shown. Appearance of an alternate water molecule to coordinate the catalytic metal site (Mg_c_/Na_c_) upon Asp427 rotation is indicated with a red arrow. Active site aspartates are shown in yellow stick representation, the incoming nucleotide in green, DNA in cyan. Mg^2+^ and Na^+^ are shown as green and purple spheres, respectively, water molecules are blue. The simulated annealing omit (F_o_-F_c_) density (green mesh) shown is contoured at 3σ, carve radius 2.0 Å. **c** Comparison of the Mg^2+^-reaction (RS_Mg_) and ground state (Ca^2+^-GS) ternary complexes. The Ca^2+^-GS complex is shown in yellow, RS_Mg_ is in magenta and are otherwise displayed as in (**b**). Ca^2+^ is shown as an orange sphere. The primer terminal (P_n_) and preceding (P_n-1_) nucleotides are indicated. The overlay was generated by superimposition of C_α_ atoms of the respective palm domains (residues 386–494).

Observing DNA polymerase catalysis in crystallo requires exchange of (activating for inactivating) metals bound to the catalytic (Ca_c_ or Na_c_) and nucleotide (Ca_n_) metal sites to initiate catalysis of nucleotide insertion^19–22,24–27^. Ca^2+^-GS ternary complex crystals were therefore transferred to a cryo-solution containing a divalent metal that supports catalysis (Mg^2+^ or Mn^2+^) and soaked at 4 °C (Fig. 2a). The crystals were frozen after increasing soak times and the structures of the resulting complexes were determined.

A 2 min soak in a Mg^2+^-containing cryo-solution generated the Mg^2+^-reaction state (RS_Mg_) ternary complex that displayed ~50% incorporation of TTP (Fig. 2b, Supplementary Table 1). Cleavage of the P_α_–P_β_ bond, inversion about P_α_, and P_α_–O3´ bond formation were evident (Fig. 2b). Apart from bond formation and cleavage, the RS_Mg_ structure was similar to the Ca^2+^-GS structure (Fig. 2c). Octahedral coordination geometry and coordination distances (2.2–2.6 Å) indicated the catalytic site was occupied by a mixture of Mg_c_ and Na_c_ (Supplementary Fig. 2a). Binding of Mg_c_ resulted in a change in the primer terminal deoxyribose sugar from C1´-exo to C4´-exo, repositioning O3´ into a catalytically compatible position (Fig. 2c, Supplementary Fig. 2a). The broad omit density surrounding P_γ_ in the GS (Fig. 1a) was not observed in RS_Mg_ (Fig. 2b, c). The altered primer sugar pucker and stabilization of P_γ_ upon metal exchange in the catalytic and nucleotide metal sites allowed P_α_ to shift closer to O3´. Asp427 could be modeled in the GS conformation and in a rotated (~90°) product conformation. This rotation was observed concurrently with loss of catalytic metal coordination upon bond formation.

Since a catalytic metal site consistent with full Mg^2+^ occupancy was not observed in the RS_Mg_ complex, we determined a structure after a shorter 1.5 min soak with Mg^2+^ (Supplementary Table 1). In this structure, ~30% bond and PP_i_ formation were observed (Supplementary Fig. 2b). The catalytic metal site displayed coordination distances (~2.2–2.3 Å), coordination geometry and electron density consistent with Mg_c_, confirming the exchange of Na_c_ for Mg_c_. These results indicate transient occupancy of Mg^2+^ at the catalytic metal site, and exchange for Na^+^ before (i.e., dissociation) and after (i.e., association) bond formation (Supplementary Fig. 2c).

### Matched product state

**Fig. 3:**
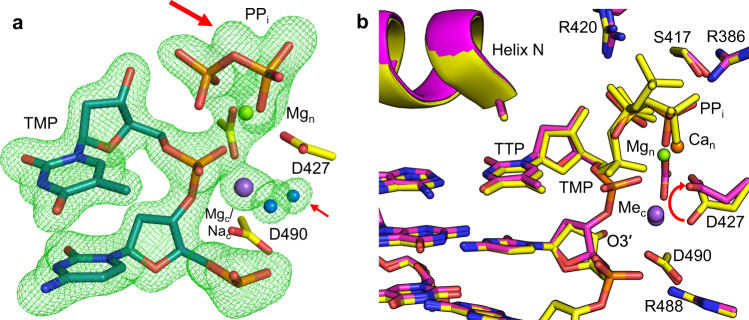
Matched Mg^2+^-product state ternary complex. **a** Mg_c_ is replaced by Na_c_ (purple sphere) upon O3´–P_α_ bond formation resulting in loss of metal coordination with Asp427 and rotation of the latter ~90° into a product conformation. Mg^2+^-dependent product formation is accompanied by partial dissociation of P1 (former P_β_ of TTP) of product PP_i_, as indicated by the appearance of simulated annealing (F_o_-F_c_) omit density (3 σ, carve radius 2.0 Å) shown by the larger red arrow. DNA is displayed in cyan stick representation, side chains are in yellow, water molecules are blue spheres. An Na_c_ coordinating water molecule is indicated by a smaller red arrow. **b** Comparison of matched Ca^2+^-GS and Mg^2+^ product complexes. Changes in the primer terminal nucleotide sugar (O3´) and rotation of Asp427 (curved red arrow) are observed. Ca^2+^-GS complex is shown in yellow, PS_Mg_ in magenta. Residues 386–494 (palm domain) of each structure were superimposed.

Complete TTP insertion and product formation was observed after a 5 min soak in the presence of Mg^2+^ as the metal ion cofactor (Fig. 3a, Supplementary Table 1). Major conformational changes were not observed in this Mg^2+^-product (PS_Mg_) complex, although subtle changes in metal and PP_i_ coordination accompanied product formation. Omit density for a bond between P_α_ and P_β_ was absent (Fig. 3a), and the active site was otherwise similar to the Ca^2+^-GS structure (Fig. 3b). Longer coordination distances and coordination geometry indicated Mg_c_ had exchanged for Na_c_, while the nucleotide metal site remained occupied by Mg_n_ (Supplementary Fig. 3a). A water molecule adopted the position of the carboxyl oxygen of Asp427 that coordinated Mg_c_ in RS_Mg_, as this side chain had adopted the product conformation (Fig. 3a, b). Loss of density for an oxygen of P1 of PP_i_ (former P_β_ of TTP), as well as appearance of additional omit density near the bridging oxygen of PP_i_, suggested an alternate PP_i_ conformation consistent with P1 dynamics (Fig. 3a, red arrow; Supplementary Fig. 3b, c). Modeling indicated the density may be partially accounted for by a component of the cryo-solution. The alternate PP_i_ conformation was removed from the final model due to weak density (Supplementary Fig. 3b, c).

### Mismatch ground state

Nucleotide insertion fidelity is a fundamental and biologically critical aspect of polymerase function. Understanding nucleotide insertion fidelity is especially important for an error-prone polymerase, such as pol λ^5,6^. A previous study^28^ determined a pre-catalytic structure of pol λ with an active site Watson–Crick-like dGMPCPP:T_t_ mismatch poised for nucleotide insertion. The truncation of loop 1 (termed pol λ DL) increased the propensity for single base substitutions and allowed structural determination of pol λ-DNA-dNTP mismatch ternary complexes^14,28^. Pol λ DL exhibited the lowest insertion fidelity for dCTP insertion opposite A_t_ in a base substitution assay^14^.

**Fig. 4:**
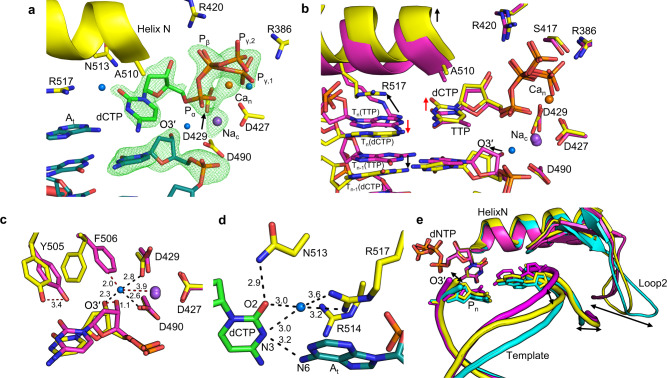
Ground state mismatch ternary complex. **a** Active site of the dCTP:A_t_ mismatch Ca^2+^-ground state. Base of incoming dCTP (green sticks) is tilted in relation to the plane of the base pair. Protein side chains are in yellow stick representation, Ca^2+^ is an orange sphere, Na^+^ a purple sphere, water molecules are blue spheres. Simulated annealing (F_o_-F_c_) omit density shown (green mesh) is contoured at 3σ, carve radius 2.0 Å. **b** Active site conformational changes in the mismatch (yellow) compared to matched (magenta) Ca^2+^-GS. Helix N is displaced relative to the matched Ca^2+^-GS, in contrast to its relative position in comparison to the binary complex (see Supplementary Figs. 1b, 4b). The palm domains (residues 386–494) of each structure were overlaid to generate the superimposition. Black arrows indicate gross structural differences, red arrows highlight differences in incoming and template nucleotide bases. **c** Conformational changes in steric gate residues (Tyr505 and Phe506), primer terminus, and appearance of a water molecule to stabilize O3´ and active site aspartates. Coordination distances (Å) are indicated with black dashed lines, atomic distances (Å) are shown with red dashed lines. Palm domains (residues 386–494) of each structure were aligned to generate the superimposition. **d** Mismatch base-pair interactions. A single hydrogen bond is observed between N3 (dCTP) and N6 (A_t_). A water molecule mediates dCTP interactions with Arg517, while Asn513 provides additional O2 (dCTP) stabilization. Hydrogen bonding (Å) is indicated with black dashed lines. **e** Overlay of binary complex (cyan) with matched (magenta) and mismatched (yellow) Ca^2+^-GS ternary complexes. Incoming nucleotide (dNTP), primer terminus (P_n_) and template coding base (opposite P_n_) are shown in stick representation. Double headed arrows indicate differences in positions of template strand, loop2 and O3´.

To provide insight into the structural basis for pol λ nucleotide insertion fidelity, we solved the Ca^2+^-GS ternary complex structure of dCTP opposite A_t_ (Fig. 4a, Supplementary Table 2). The nucleotide binding site is fully occupied by dCTP, as indicated by the simulated annealing omit (F_o_-F_c_) density map. The active site is in a conformation inconsistent with product formation (Fig. 4a, b), as O3′ lies even further (~6 Å, Supplementary Fig. 4a) from P_α_ than in the matched Ca^2+^-GS complex (~5 Å, Fig. 1c). Active site metals (Na_c_ and Ca_n_), catalytic aspartates (Asp427, Asp429 and Asp490), and triphosphate stabilizing residues (Arg318, Arg420, Ser417) overlay identically with their respective positions in the matched Ca^2+^-GS complex (Fig. 4b). Extended density surrounding P_γ_ is again observed suggesting alternate conformations (Fig. 4a). A novel water molecule coordinates O3′ and the carboxylate oxygens of Asp429 and Asp490 (Fig. 4a–c, Supplementary Fig. 4b). The hydroxyl of Tyr505 forms a hydrogen bond with N1 of A_t_ and is ~3.4 Å from its position in the matched Ca^2+^-GS complex (Fig. 4c, Supplementary Fig. 4b). The aromatic ring of Phe506 is positioned proximal rather than parallel to that of Tyr505 observed in the matched Ca^2+^-GS. Stabilization by Phe506 wedges Tyr505 between the template adenine and incoming cytosine bases that both rotate out of the plane of the matched base-pair (Fig. 4b, red arrows), preventing adoption of Watson–Crick-like base-pair geometry. Only a single hydrogen bond is thus inferred between N3 of incoming dCTP and N6 of A_t_ (Fig. 4d). This observation differs from the dGMPCPP:T_t_ mispair that displayed cognate Watson–Crick base-pair geometry^28^. The tilting of the cytidine base towards Ala510 has resulted in a shift of α-helix N by ~1.5 Å from its position in the matched Ca^2+^-GS or binary complexes. Thus, unlike the matched Ca^2+^-GS complex, Arg517 now stabilizes A_t_ and interacts with O2 and N3 of dCTP through a unique water molecule. Interactions with Asn513 and Ala510 confer additional dCTP stabilization (Fig. 4a, b, d). Arg514 has shifted from stabilizing A_t_ to pointing away from it, into a position where it may coordinate a backbone phosphate of the template strand (Fig. 4d, Supplementary Fig. 4c).

The mismatched Ca^2+^-GS ternary complex displays characteristics not observed in either the binary or the matched Ca^2+^-GS ternary complex. Altered conformation and positioning of the lyase domain, displacement of the main chain backbone from Tyr505 to Trp573, and associated side chain rearrangements are observed compared to the matched Ca^2+^-GS (Fig. 4e, Supplementary Fig. 4b, d, e). Mobile loop 2 is nearer the downstream template strand in the matched relative to the mismatched Ca^2+^-GS complex (Fig. 4e, Supplementary Fig. 4d, e). These differences modulate template strand positioning to place the coding templating base ~3.5 Å from its position in the matched Ca^2+^-GS complex. The coding template base thus provides sub-optimal interactions with the incoming nucleotide (Fig. 4a, b, d, e). Altered primer terminal base pair positioning therefore repositions O3′ ~1.1 Å away from its position in the matched Ca^2+^-GS complex (Fig. 4b, c). Overall, the mismatch Ca^2+^-GS ternary complex is grossly distorted compared to the equivalent matched ternary complex.

### Mismatch incorporation

**Fig. 5:**
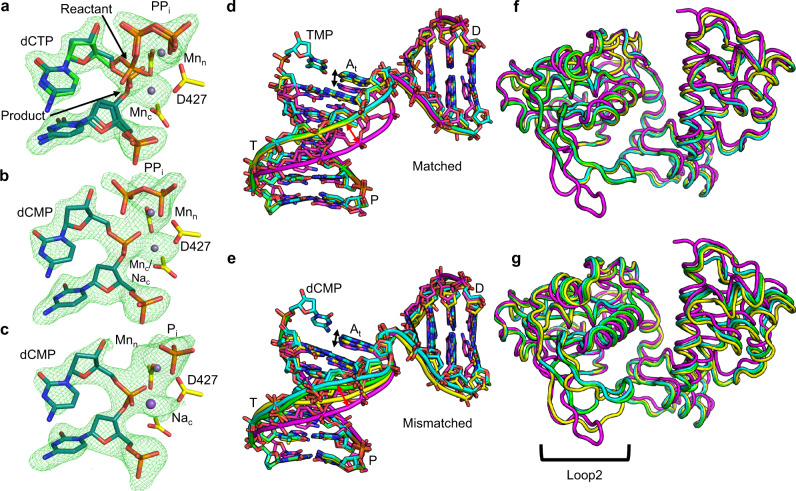
Mismatch incorporation. **a** Mismatch Mn^2+^-reaction state ternary complex after a 225 min soak. **b** Mn^2+^-mismatch product state (PS_Mn_) after a 420 min soak. **c** Extended soak of the Mn^2+^-mismatch product (PS_Mn_^ES^) complex. In (**a**), (**b**) and (**c**) dCTP is shown in green stick representation, DNA in cyan, sidechains are yellow. Smaller magenta spheres are Mn^2+^, the large purple sphere is Na^+^. Simulated annealing omit (F_o_-F_c_) density is shown as a green mesh contoured at 2.5 σ, carve radius 2.0 Å. Global conformational changes in DNA during (**d**) matched and (**e**) mismatched insertion. Structural overlays of the binary complex (magenta), Ca^2+^-GS (yellow), RS_Mn_ (cyan), PS_Mn_ (green) are shown. Template (T), primer (P) and downstream (D) strands are indicated. DNA phosphate backbone is shown as a tube, nucleotides are shown in stick representation. Global conformational changes in protein backbone during matched (**f**) and mismatched (**g**) nucleotide insertion. A bracket highlights loop 2 of the protein.

Use of Mn^2+^ as the divalent metal ion increases the binding affinity of nucleotides to the active site and allows in crystallo mismatch incorporation by pol β^20^. Mn^2+^ anomalous diffraction additionally allows identification of stably bound Mn^2+^ atoms in x-ray crystal structures^18–22,24,29^. We therefore applied the time-lapse approach using either Mg^2+^ or Mn^2+^ as the divalent metal ion cofactor with mismatched incoming dCTP opposite A_t_. Occupancy refinement of the mismatch reaction state (RS) ternary complexes indicated ~50% insertion had occurred after 120 min and 225 min soaks in cryo-solutions containing Mg^2+^ (RS_Mg_; Supplementary Fig. 5a, Supplementary Table 2) or Mn^2+^ (RS_Mn_; Fig. 5a, Supplementary Table 3), respectively. Despite active site distortion, exchange of Na_c_ and Ca_n_ for Mg^2+^ or Mn^2+^ in the catalytic and nucleotide metal sites, and a concomitant change in sugar pucker, reposition O3′ into a position suitable for chemistry (Fig. 5a, Supplementary Fig. 5b). The mismatch product structures were obtained after 300 min (Mg^2+^, PS_Mg_; Supplementary Fig. 5c) and 420 min (Mn^2+^, PS_Mn_; Fig. 5b) soaks. Complete product formation had occurred and the phosphates of PP_i_ were in positions expected directly after bond cleavage. Na_c_ had partially replaced Mg_c_ in the catalytic metal site and Asp427 had rotated ~90° into a product conformation (Supplementary Fig. 5c). Mn_c_ remained bound and Asp427 was observed in only the GS conformation (Fig. 5b). Extending the soaks to 960 min with either metal resulted in structures similar to the PS complexes (Fig. 5c, Supplementary Fig. 5d). Na_c_ was bound to the catalytic metal site in both reactions as confirmed by the lack of anomalous density for Mn^2+^ (Supplementary Fig. 5e). The nucleotide metal site was still occupied by Mg_n_ (Supplementary Fig. 5d) or Mn_n_ (Fig. 5c) and Asp427 occupied the product conformation in these longer soaks. PP_i_ had partially dissociated with both metals (Fig. 5c, Supplementary Fig. 5d).

Comparison of matched and mismatched structural intermediates reveals a pol λ fidelity checkpoint during and after nucleotide insertion. The matched Ca^2+^-GS ternary complex supports efficient insertion as matched reaction intermediates overlay identically with the Ca^2+^-GS ternary complex (Fig. 5d, f). Mismatch insertion occurs through an altered reaction trajectory where local and global conformational changes accommodate the distorted active site to enable inefficient mismatch insertion (Fig. 5e, g). The different insertion trajectories result in distinct product complexes (Supplementary Fig. 5f) that are nevertheless more similar than the GS-complexes (Supplementary Fig. 4e). That mismatch insertion can proceed from a distorted ground state without first assuming the canonical ‘closed’ conformation is an unexpected finding^12,28^.

### The product metal

The Mg^2+^-reaction (RS_Mg_) and -product (PS_Mg_) complexes lacked density in the region of the recently discovered product metal during matched insertion, reminiscent of pol μ catalyzed matched TTP insertion^22^. In the latter case, use of Mn^2+^ as the divalent metal ion cofactor, instead of Mg^2+^, permitted discovery of the third or product metal. We therefore applied the time-lapse approach using Mn^2+^ as the metal ion cofactor to observe matched incoming TTP insertion opposite A_t_ (Fig. 2a).

**Fig. 6:**
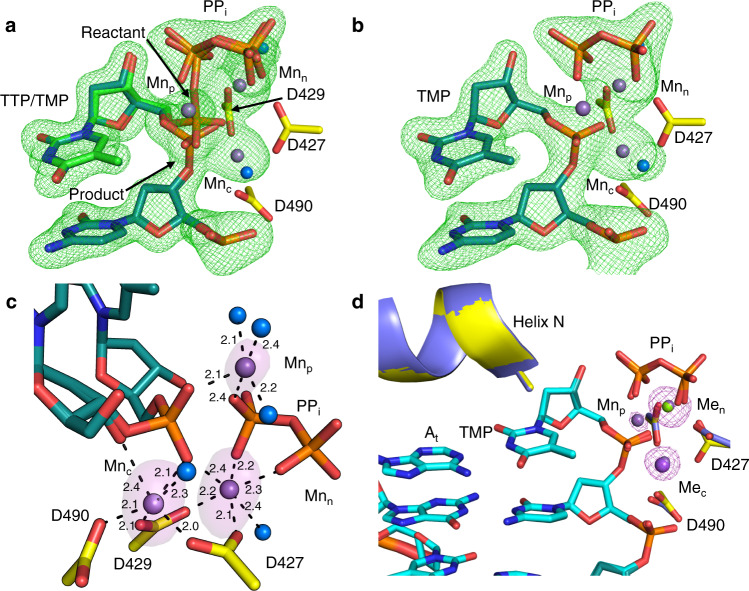
Manganese-mediated insertion and the product metal. **a** Matched (TTP:A_t_) Mn^2+^-reaction state (RS_Mn_) complex after a 5 min soak. Partial nucleotide insertion with inversion about P_α_ and concurrent generation of PP_i_ are evident from the simulated annealing (F_o_-F_c_) omit density shown at 3 σ (green mesh), carve radius 2.0 Å. Product formation is accompanied by the appearance of an additional metal (Mn_p_). Active site aspartates are shown in yellow stick representation, the incoming nucleotide is in green, DNA in cyan. Mn^2+^ atoms are shown as magenta spheres, water molecules in blue. **b** Matched Mn^2+^-product state after a 20 min soak. Mn_p_ is still present (F_o_-F_c_ density omitted for clarity). Simulated annealing omit (F_o_-F_c_) density (green mesh) shown is contoured at 3 σ, carve radius 2.0 Å. **c** Active site metal coordination and anomalous density in the Mn^2+^-reactant state. Anomalous density is shown as a magenta surface at 5 σ, carve radius 2.0 Å. Metal coordination is shown with dashes, coordination distances (Å) are indicated. dCTP was omitted for clarity. **d** Structural overlay of the Mg^2+^ (purple protein, light cyan DNA) and Mn^2+^ (yellow protein, dark cyan DNA) product complexes. Anomalous density (magenta mesh) is shown contoured at 5 σ, carve radius 2.0 Å.

The Mn^2+^-reaction (RS_Mn_) and product (PS_Mn_) states of TTP:A_t_ insertion were observed after 5 min (Fig. 6a, Supplementary Table 4) and 20 min (Fig. 6b) of soak in a cryo-solution containing Mn^2+^. An additional active site metal, with strong anomalous density, was observed in both structures (Fig. 6a–d). This metal coordinated product phosphate oxygens of the nascent primer terminus, an oxygen of P1 of PP_i_ (former P_β_ of TTP), and three water molecules. Since the metal coordinated two product oxygens and was absent in the Ca^2+^-GS, we denote the metal as the product metal (Mn_p_). All three metal binding sites in both structures were occupied by Mn^2+^ (Fig. 6a–d). RS_Mn_ and PS_Mn_ were otherwise identical to the corresponding Mg^2+^ complexes (Fig. 6d).

The observed rate of DNA synthesis in crystallo is slower than observed in solution likely due to the restraining nature of the crystalline environment and the low temperature (4 °C) employed. Single-turnover kinetic analyses (enzyme >> gapped DNA) of nucleotide insertion with Mg^2+^ (Supplementary Fig. 6a, Supplementary Table 5) or Mn^2+^ (Supplementary Fig. 6b) as the metal cofactor were performed to determine the nucleotidyl transferase efficiency of pol λ. The observed rate constant (*k*_pol_) for TTP insertion opposite template A_t_ in a single-nucleotide gapped DNA substrate was similar to values reported previously^6,14,30^. The efficiency of TTP insertion was ~3-fold higher with Mn^2+^ than with Mg^2+^ (Supplementary Table 5).

To further evaluate the impact of the product metal on catalysis by pol λ, we made use of the phosphorothioate analogue of TTP (TTPαS)^31^. Phosphorothioate nucleotide analogues, where sulfur substitutes a non-bridging oxygen of the α-phosphate, have previously been employed to decipher the functional role of metal coordination on DNA polymerase catalysis^21,22,32^. Mn^2+^ is generally thio-phobic preferring to bind oxygen rather than sulfur ligands^33^. As shown in Supplementary Fig. 6c (Supplementary Table 4), the Ca^2+^-GS ternary complex of TTPαS opposite A_t_, determined to 1.82 Å resolution, was identical to the matched insertion Ca^2+^-GS complex. Ca^2+^-GS crystals of the TTPαS:A_t_ insertion were then soaked in cryo-solutions containing Mn^2+^. The product state was obtained after a 60 min soak (Supplementary Fig. 6d, Supplementary Table 4). Active site interactions remained identical to matched (TTP) insertion. Anomalous or omit density corresponding to a product metal was not observed in any structures determined with Mn^2+^ and TTPαS.

### Pyrophosphate release

Post-chemistry steps in the DNA polymerase catalytic cycle may influence the forward and reverse reactions but are poorly characterized. To observe PP_i_ and product metal release, we performed extended soaks of the product complexes. The 60 min soak structures (Fig. 2, PS*; Supplementary Fig. 7a, b, Supplementary Tables 1, 4) displayed full TTP incorporation. The catalytic and nucleotide metal sites were occupied by Na_c_ and Mg_n_ in the Mg^2+^ soak (PS*, Supplementary Fig. 7a, middle), whereas these sites remained occupied by Mn^2+^ in the Mn^2+^ soak (PS*, Supplementary Fig. 7b, middle). The Mn^2+^ product metal was still observed after 60 min, as confirmed by the presence of an anomalous signal, and occupancy refinement indicated it had partially dissociated (~40%). The product conformation of Asp427 was observed in the 60 min Mg^2+^ soak (PS*, Supplementary Fig. 7a, middle), while this residue could be modeled in both ground and product conformations in the Mn^2+^ soak (Supplementary Fig. 7b, middle). Additional omit density in the Mg^2+^ structure was still observed near the bridging oxygen of PP_i_ (PS*, Supplementary Fig. 7a, red circle), and density in the same location for an alternate conformation of P1 of PP_i_ (former P_β_ of TTP) appeared in the 60 min Mn^2+^ soak (PS*, Supplementary Fig. 7b, red circle).

Structures after the 960 min soaks (PS_Mn_^ES^ and PS_Mg_^ES^) also displayed full turnover and were nearly identical to the structures after the 60 min soaks (Supplementary Fig. 7a, b, Supplementary Tables 1, 4). Weaker overall density indicates PP_i_ had dissociated from the active site in the 960 min Mg^2+^ soak (PS_Mg_^ES^, Supplementary Fig. 7a, right). Density for P2 of PP_i_ (former P_γ_ of TTP) remained in the 960 min Mn^2+^ soak, suggesting that PP_i_ was retained in the active site (PS_Mn_^ES^, Supplementary Fig. 7b, right). Additional density near the bridging oxygen of PP_i_ was now present in the Mn^2+^ soak (PS_Mn_^ES^, Supplementary Fig. 7b, right, red circle). Density for P1 (former P_β_ of TTP) was substantially reduced in structures determined from both soaks with both metals (PS_Mn_^ES^ and PS_Mg_^ES^; Supplementary Fig. 7a, b, right). While metal coordination was otherwise identical to the PS and PS* structures with either metal, lack of anomalous density surrounding the product metal site indicates the product metal had dissociated from the active site in the 960 min Mn^2+^ soak (PS_Mn_^ES^, Supplementary Fig. 7b, right).

## Discussion

Identification of molecular intermediates along the catalytic cycles of right and wrong nucleotide insertion is required to understand polymerase fidelity in DNA replication and repair. We developed a high-resolution time-lapse crystallography approach to capture snapshots of DNA polymerase λ undergoing insertion of natural nucleotide substrates. Previously hidden polymerase fidelity checkpoints are uncovered that hasten insertion of the right nucleotide and deter insertion of the wrong nucleotide.

### “Sensing” the right or wrong nucleotide

**Fig. 7:**
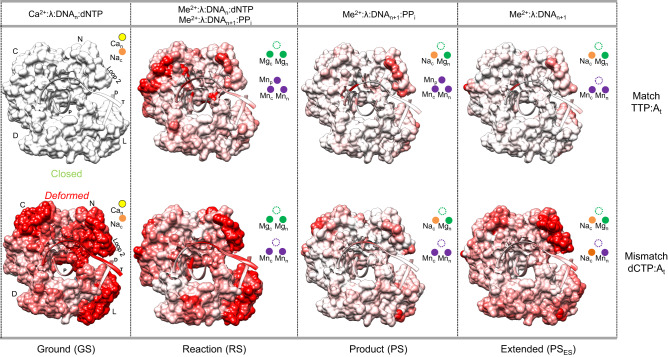
Hidden pol λ fidelity mechanisms. Panels display structural intermediates corresponding to the stage of nucleotide incorporation indicated below. Ligands observed at each stage of reaction are indicated above the central panels. “Closed” and “Deformed” refer to the state of the polymerase in matched and mismatched insertion, respectively. Regions of the protein or DNA shaded from white (0 Å) to red (1 Å and above) display differences in positions of backbone C_α_ atoms compared to the previous intermediate (RS compared to GS, PS compared to RS, and PS_ES_ compared to PS). Shading of the mismatched ground state complex from white (0 Å) to red (1 Å and above) reflects differences in C_α_ positions compared to the matched ground state ternary complex. The catalytic (Me_c_), nucleotide (Me_n_) and product metals (Mn_p_) are indicated by spheres colored according to metal identity (Ca^2+^, yellow; Na^+^, orange; Mg^2+^, green; Mn^2+^, magenta). Protein subdomains are labeled as the N (thumb), C (palm), D (fingers) and L (lyase) domains. Superimposition and shading was performed with the program Chimera by overlaying the palm domains (residues 386–494) of each structure.

The high-resolution structural intermediates captured along matched and mismatched natural nucleotide insertion trajectories reveal a “nucleotide-sensing” mechanism that enables conformational alignment of the polymerase-substrate complex to modulate insertion in response to active site base pair geometry. Matched nucleotide induced local and global conformations mediate optimal alignment of active site structure, template strand and catalytic residues, positioning the coding template base for efficient insertion^12^. Steric gate aromatic residues Tyr505 and Phe506 stabilize the matched incoming nucleotide, adopting shifted and flipped positions, respectively, that interfere with the optimal geometry of the incoming and template nucleotides in the mismatched Ca^2+^-ground state ternary complex (Fig. 4, Supplementary Fig. 4). A shifted α-helix N enables Arg517 to stack over the template base in the mismatched ground state, instead of stabilizing the template strand in the minor groove of the matched ternary complex, as is seen with the corresponding pol β residue, Arg283 (Fig. 4b, d)^34^. Arg514 alters conformation in mismatch insertion offering decreased stabilization of the template strand during insertion (but not in the GS complex), instead of stacking with the template base to stabilize the matched template strand (Supplementary Fig. 4c). Mismatch induced active site adjustments alter the conformations of α-helix N, the C-terminal domain from Tyr505 to Trp573 (including loop 2), and the lyase domain (Figs. 4b, e, 7, Supplementary Fig. 4b, d, e). These changes reposition the template and primer strands along with O3′ inducing a catalytically suboptimal polymerase conformation that allows inefficient mismatch insertion. The distorted base pair geometry exposes O3′ for coordination by a novel water molecule in the space vacated by the mismatched conformation of Phe506 that may serve as a general proton acceptor during catalysis (Fig. 4a–c, Supplementary Fig. 4a, b). This water molecule, that has not been previously observed in X-family polymerases, may allow O3′ deprotonation to promote inefficient mismatch insertion^35,36^. Template strand repositioning combined with global protein conformational adjustments together constitute a previously hidden pol λ fidelity checkpoint.

Reaction intermediates along the matched and mismatched insertion trajectories revealed that the distorted mismatched base pair induced global conformational adjustments during misinsertion but minimally during more optimal matched insertion (Fig. 7). The more similar product conformations (Figs. 5d–g, 7, Supplementary Figs. 4e, 5f) generated by this catalytic induced fit may modulate PP_i_ release and the susceptibility to undergo the reverse chemical reaction, thus influencing fidelity. Previously hidden checkpoints during nucleotide insertion and in the product complex therefore modulate pol λ nucleotide insertion fidelity.

### Purine-pyrimidine mismatch insertion lacking Watson–Crick geometry

Watson and Crick proposed that spontaneous transition mutations involve rare tautomeric forms of nucleotide bases^37,38^. Tautomeric and anionic forms of purine-pyrimidine mismatches can adopt Watson–Crick-like geometry in the polymerase active site^28,39–42^ that may permit a mismatch to avoid polymerase fidelity checkpoints^28,43^. Pre-catalytic complexes of the dCTP:A_t_ mismatch (with Mn^2+^ but not Mg^2+^) for the *Bacillus* fragment^42^, dTMPPNP:G_t_ mismatch (with Mn^2+^) for pol β^44^, and dGMPPCP:T_t_ mismatch (with Mn^2+^) for pol λ^28^ displayed Watson–Crick-like geometry. A primer terminal pol λ G:T mispair displayed a wobble conformation^28^.

Our structural intermediates show that the purine–pyrimidine (dCTP:A_t_) mismatch is accommodated in a non-tautomeric distorted wobble-like conformation coplanar with the coding template base (Fig. 4a, b, d). The dCTP:A_t_ mismatch is thus distinct from the pre-catalytic dGMPPCP:T_t_ mismatch and the matched equivalent, as well as that observed in pol β and the *Bacillus* fragment. The lack of Watson–Crick geometry was identically maintained throughout dCTP:A_t_ misinsertion in the presence of either Mg^2+^ or Mn^2+^ (Fig. 5a–c, Supplementary Fig. 5a, c, d). The distorted base pair geometry promotes misinsertion through an altered reaction trajectory compared to matched insertion (Figs. 5d–g, 7). Unexpectedly, the catalytic mechanism for C-A (dCTP:A_t_) misinsertion starkly contrasts with the G-T and matched insertions.

The “protein-stabilized” mismatched base interaction observed here is reminiscent of that in Y-family polymerase Rev1^45^. In Rev1, Arg324 hijacks base-pairing interactions with incoming dCTP by displacing the template base outside of the double helix. In pol β, Arg283 (pol λ Arg517 equivalent) stabilizes the dCTP mismatch product complex^46^. Recognition and stabilization of the wrong base in a near template-independent manner is a pol λ fidelity checkpoint.

### Catalysis and metal dynamics

Positioning of catalytic residues and P_α_, as well as metal coordination were near identical in matched and mismatched Ca^2+^-GS complexes regardless of template strand positioning (Fig. 4b). O3´ was shifted in the mismatch (Fig. 4b, c) and did not coordinate the catalytic metal. Unlike the nucleotide metal site, the catalytic metal site thus does not bind Ca^2+^ in either matched (Fig. 1a, c) or mismatched (Fig. 4a, Supplementary Fig. 4a) Ca^2+^-GS ternary complex and accommodates Na_c_ instead. Upon metal exchange, binding of a divalent metal ion (e.g., Mg^2+^ or Mn^2+^) to the catalytic metal site alters the conformation of the nucleotide deoxyribose by coordinating O3´ and facilitating its deprotonation at physiological pH^47^. The altered primer terminus sugar pucker shifts P_α_(dNTP) into a position compatible with catalysis. The shift in nucleotide sugar pucker is hampered in the mismatch by a novel O3′ coordinating water molecule (Fig. 4a–c, Supplementary Fig. 4a, b) that may hinder its deprotonation to delay in-line attack at P_α_ in early misinsertion.

Our structural intermediates more generally indicate that the geometry of the nascent base pair directs the metal dynamics that modulate catalysis and fidelity, especially during and after insertion. Unstable coordination of Mg_c_ in the matched insertion (Supplementary Fig. 2c) facilitates rapid Mg_c_ release and exchange with Na_c_ upon bond formation (Supplementary Figs. 2a, 3a). Since a divalent metal in the catalytic metal site is required for the reverse reaction^48,49^, and considering the increased stability of octahedrally coordinated Mg_c_ in mismatch insertion (Supplementary Figs. 5b, 8a), rapid catalytic metal release is expected to promote DNA synthesis fidelity.

The product metal (Mn_p_) was discovered to transiently bind matched but not mismatched product phosphate oxygens with Mn^2+^, but not Mg^2+^, as the metal ion cofactor (Fig. 6a–d, Supplementary Fig. 7b). Mn_p_ was thus observed to stabilize the matched but not mismatched product complex and promote DNA synthesis fidelity likely through inhibition of the reverse (chemical) reaction^48,49^. Unlike for pol β^20^, Mn_c_ remains bound throughout matched insertion but dissociates and is replaced by Na_c_ in the mismatched post-catalytic soak of the product complex (Fig. 5c, Supplementary Fig. 5e). In the absence of Mn_p_, delayed Mn_c_ release thus likely promotes inefficient DNA synthesis. The delayed loss of Mn_c_ from the mismatched product complex in the absence of Mn_p_, but not from the matched product complex in the presence of Mn_p_, also indicates that base pair geometry modulates polymerase fidelity by influencing the coordination of the catalytic metal site in the product complex (Fig. 6b, c, Supplementary Fig. 7b). This is likely a more general feature of DNA polymerase fidelity. Additionally, the global “induced fit” during catalysis may promote generation of a product conformation capable of Mn_c_ release.

The carboxyl oxygens of Asp427 coordinate both catalytic and nucleotide metals in the ground state ternary complex. Mg^2+^-mediated catalysis (RS_Mg_ to PS_Mg_^ES^) is accompanied by a ~90° rotation of Asp427 carboxyl oxygens into a conformation in which only one oxygen coordinates one or both metal sites (Supplementary Fig. 7a). Mg_c_ release and exchange for Na_c_ is observed concurrently with this rotation and a water molecule binds to replace lost coordination with Asp427 (Supplementary Figs. 2a, 3a). Since Mn_c_ remains bound throughout the reaction, the rotation of Asp427, observed during product release steps of the Mn^2+^ reaction (PS_Mn_* and PS_Mn_^ES^; Supplementary Fig. 7b), suggests that the rotation of Asp427 is likely uncoupled from Mn_c_ release and may reflect differing electronic features of these reactions. A similar rotation of the equivalent aspartate, Asp330, was observed during catalysis by pol μ^22^. In contrast, rotation of the corresponding aspartate, Asp190, was not observed during pol β catalysis^20^.

The nucleotide metal site remains occupied long after catalysis with both metals is complete (Fig. 5c, Supplementary Figs. 5d, 7a, b). The presence of Mg^2+^ or Mn^2+^ in the nucleotide metal site is required to stabilize P_γ_ and enable P_α_ to reposition for in-line attack by O3′ (Figs. 1a, b, 4a, Supplementary Fig. 1a). Stabilization and positioning of P_γ_ may thus be an important feature of catalysis.

### Product metal

The third or product metal (Mn_p_) was observed to transiently bind the active site after chemistry (Fig. 6a, b). The product metal associates with the pol λ active site in the matched Mn^2+^ reaction state ternary complex (Figs. 6a, 7, Supplementary Fig. 8a–c). In contrast, the product metal is not observed with Mg^2+^, in mismatch insertion with either metal (Fig. 5a–c, Supplementary Fig. 5a, c, d), or in TTPαS^21,48^ insertion (Supplementary Fig. 6c, d). Kinetic analysis in the presence of Mg^2+^ or Mn^2+^ revealed similar insertion efficiencies with both metals (Supplementary Fig. 6a, b, Supplementary Table 5). Lack of Mg_p_ and looser Mn_p_ coordination may indicate mechanistic differences in pols λ and μ^22^ compared to pols β^20^ and η^21,24^. Overall, our observations suggest that the product metal promotes polymerase insertion of base pairs exhibiting Watson–Crick-like geometry but is not essential for DNA synthesis.

To further evaluate the effect of the product metal, an oxygen of P_α_(TTP), that upon matched nucleotide insertion would bind the product metal, was substituted with sulfur (Supplementary Fig. 6c). While the product metal was not observed in this Mn^2+^-mediated reaction (Supplementary Fig. 6d)^21,22^, the rate of turnover *in crystallo* was decreased likely due to the weaker electron withdrawing ability of sulfur, rendering P_α_ less reactive and influencing the properties of the catalytic site, or due to steric differences^50^.

The product metal has been observed to coordinate product oxygens of the *in crystallo* DNA synthesis reactions of X-family pols β^19,20,48,49,51^ and μ^22^, as well as Y-family pols η^21,24^ and *E. coli* Pol4 (DinB)^23^. In these enzymes, the product metal is released after bond formation prior to PP_i_ release from the active site. Active site positioning of the product metal is similar in the X-family polymerases (Supplementary Fig. 8b, c).

### Pyrophosphate release

Polymerase PP_i_ release involves altered active site protonation and conformational adjustments of PP_i_ (pol µ)^22,52^ or a subdomain (pol β)^20,22^. In contrast to pols μ^22^ and β^20^, P1 of PP_i_ (former P_β_ of TTP) dissociates first in pol λ, as suggested by the progressive loss of electron density in both matched and mismatched insertions (Fig. 5a–c, Supplementary Figs. 3b, c, 5a–d, 7a, b). Loss of density for P1 coincides with loss of Mn_p_, while density for P2 remains throughout the reaction, suggesting that product metal release modulates PP_i_ release. The observed PP_i_ conformational changes are similar in the matched Mg^2+^ and Mn^2+^ intermediates but delayed in the presence of Mn_p_. Increased P1 dynamics is associated with exchange of Mg_c_ for Na_c_ that may promote PP_i_ release due to disruption of an active site bimetallic bond^53^. Mn_c_ release in mismatch insertion may thus similarly be required to facilitate PP_i_ release since the product metal is absent. P1 dynamics in the absence of Mn_p_ thus promotes fidelity as PP_i_ is unavailable to undergo the reverse reaction, where P1 of PP_i_ (former P_β_ of TTP) reacts with the primer terminal phosphate generating an intact triphosphate and a primer that is one nucleotide shorter^32^. Post-catalytic conformational changes in the mismatched but not matched product complex suggest PP_i_ release may require conformational changes (Fig. 7). Similarly to pol μ^22^, Mg_n_ and Mn_n_ remain bound to the active site upon PP_i_ release, indicating that PP_i_ and nucleotide metal release are uncoupled. Active site water molecules likely complete the Me_n_ coordination sphere after PP_i_ dissociation.

## Methods

### Protein expression and purification

Truncated human pol λ (residues 242–575) with modified loop1^14^ (residues 463–471 replaced with sequence KGET). The construct included a C543A mutation to improve crystallizability. Pol λ was overexpressed in BL21(DE3)CodonPlus-RIL cells (Invitrogen) and purified as follows^54^. Cells were lysed by sonication in Lysis Buffer (25 mM Tris pH 7.5 (25 °C), 350 mM NaCl, 1 m M DTT, 1 mM EDTA) and 0.1% polyethylene-imine (PEI, %v/v) was added to precipitate nucleic acids. The clarified supernatant was incubated in-batch with Q Sepharose FF resin (Cytiva) and pol λ was captured from the supernatant on a Heparin HiTrap HP column (Cytiva). After elution with a linear gradient of Elution Buffer (25 mM Tris pH 7.5 (25 °C), 1 M NaCl, 1 mM DTT, 1 mM EDTA), the eluate was dialyzed overnight at 4 °C into Storage Buffer (25 mM Tris pH 7.5 (25 °C), 100 mM NaCl, 1 mM DTT). After ion exchange chromatography (MonoS HR 10/10, Cytiva) with a linear gradient of Elution Buffer, the eluate was dialyzed into Storage Buffer. Size-exclusion chromatography was then performed on a HiLoad Superdex 200 26/600 gel filtration column (Cytiva) in Storage Buffer. Pol λ was again dialyzed, concentrated to 16 mg/ml and stored at −80 °C after flash freezing in liquid nitrogen.

### DNA preparation

For crystallization, an 11-mer template oligonucleotide (5′- CGGC**A**GTACTG−3′, template base in **bold**, Supplementary Table 6) was annealed with a 6-mer upstream (5′-CAGTAC-3′) oligonucleotide and a 5′-phosphorylated downstream 4-mer (5′-pGCCG-3′) oligonucleotide in a 1:1:1 ratio to create a duplex DNA with a single nucleotide gap. Oligonucleotides were dissolved in 100 mM Tris-HCl pH 7.5, heated to 95 °C for 5 mins and then cooled down to 4 °C at a rate of 1 °C/min. For kinetic studies, the sequences of the oligonucleotides were: 5′-CGG TGA TAT GCA GTC AGT AC-3′ (primer strand, Supplementary Table 6); 5′-pGCC GAG CGT CAA TG-3′ (downstream strand); 3′-GCC ACT ATA CGT CAG TCA TG**A** CGG CTC GCA GTT AC-5′ (template strand, template base in **bold**). The primer oligonucleotide had a 5´-6-carboxyfluorescein label, and the downstream oligonucleotide was 5´-phosphorylated. The coding nucleotide in the template sequence is in bold. Oligonucleotides were resuspended in 10 mM Tris-HCl (pH 7.4), 1 mM EDTA and oligonucleotide concentrations were determined at 260 nm using extinction coefficients provided by Integrated DNA Technologies. A one-nucleotide gapped DNA substrate was prepared by annealing primer with 20% excess downstream and template oligonucleotides (1:1.2:1.2 molar ratio). The annealing reactions were performed in a thermal cycler by heating at 95 °C for 5 min followed by cooling to 10 °C (1 °C/min).

DNA was purchased PAGE purified for crystallography and HPLC purified for kinetics from Integrated DNA Technologies (Coralville, IA).

### Time-lapse crystallography

Pol λ ternary complex crystals were grown by mixing protein (16 mg/ml) with annealed DNA in a 1:2 ratio and incubated at 4 °C for 2 h. After addition of 2 mM dNTP and 10 mM CaCl_2_, the mixture was incubated for an additional 2 h on ice. Crystallization plates were set up by mixing pol λ-DNA-dNTP ternary complex with well solution (20 mM bicine pH 7.5, 14–20% PolyPure PEG, 300 mM Na-K tartrate) in sitting drop format. Time-lapse crystallography was performed as follows: after a short pre-soak wash, ternary complex crystals were transferred to a drop consisting of artificial mother liquor (10 mM bicine pH 7.5, 20% PolyPure PEG, 75 mM Na-K tartrate, 20% ethylene glycol) supplemented with 10 mM CaCl_2_, 50 mM MgCl_2_ or 50 mM MnCl_2_ for varying times. The reaction was stopped by plunging the crystal into liquid nitrogen.

### Data collection and refinement

Data collection was performed at the Advanced Photon Source (Argonne National Laboratory, Chicago, IL) on the ID22 or BM22 beamlines (SER-CAT, Southeast Regional Collaborative Access Team) using the Mar300HX area detector at 1.00 Å. Data were processed and scaled using the program HKL2000^55^ or HKL3000^56^. The initial model of the ground state ternary complex was determined using molecular replacement with a previously determined structure of pol λ (PDB id 3UPQ^54^). Refinement was carried out using the PHENIX software package and iterative model building was performed using Coot^57^. Utilizing the R_free_ rejection set from the starting model (PDB ID 3UPQ), partial catalysis models were generated with both the reactant and product species with occupancy refinement. The Ca^2+^-bound ground state (GS) ternary complex structure was then used as the starting model to solve other structures. Metal coordination and B-factors were verified using the CheckMyMetal Server^58^. Simulated annealing (F_o_-F_c_) omit density maps were generated by deleting the regions of interest and performing simulated annealing with harmonic restraints. Unless otherwise noted, simulated annealing (F_o_-F_c_) omit and anomalous maps are contoured at 3 and 5 σ, respectively, with a carve radius of 2.0 Å. Ramachandran analysis determined at least 97% of residues lie in allowed regions. The figures were prepared in PyMol (Schrodinger).

### Kinetic assays

Single-turnover kinetic assays were employed to measure the rate of the first insertion (*k*_pol_) and the apparent equilibrium nucleotide dissociation constant (*K*_d,app_). The assays were performed on a Kintek RQF-3 chemical quenched-flow apparatus (KinTek Corp., Austin, TX). Truncated pol λ with modified loop1 (1 μM) was pre-incubated with single-nucleotide gapped DNA substrate (100 nM) and mixed with varying concentrations of metal-TTP in 50 mM Tris-HCl, pH 7.4 (37 °C), 100 mM KCl, 10% glycerol, 100 μg/ml bovine serum albumin, 1 mM dithiothreitol, and 0.1 mM EDTA. Mg^2+^ or Mn^2+^ was added so that 10 mM Mg^2+^ or 1 mM Mn^2+^ free metal was present. The reaction was quenched with 0.25 M EDTA and reaction products mixed with an equal volume of formamide dye. After separation on 18% denaturing gels, products were quantified using a Typhoon phosphorimager and Imagequant software. The secondary plots of dNTP dependencies on the observed single-exponential rates were fit to a hyperbolic equation to derive kinetic parameters. The double exponential behavior observed at higher [dNTP] was not included in the fit.

### Reporting summary

Further information on research design is available in the Nature Research Reporting Summary linked to this article.

## Supplementary information

Supplementary Information

Peer Review File

Reporting Summary

## Data Availability

The data that support this study are available from the corresponding authors upon reasonable request. The coordinates and structure factors for the reported crystal structures have been deposited in the Protein Data Bank (PDB) under the following accession numbers: 7M43, 7M44, 7M45, 7M46, 7M47, 7M48, 7M49, 7M4A, 7M4B, 7M4C, 7M4D, 7M4E, 7M4F, 7M4G, 7M4H, 7M4I, 7M4J, 7M4K, 7M4L. Source data are provided as a Source Data file.

## Source data

Source Data
