# Supplementary material for: Watching right and wrong nucleotide insertion captures hidden polymerase fidelity checkpoints
Source: Nat Commun. 2022 Jun 9;13:3193. doi: 10.1038/s41467-022-30141-w (PMC9184648; doi:10.1038/s41467-022-30141-w)
Supplement: Supplementary file 1 — Supplementary Information [file 41467_2022_30141_MOESM1_ESM.pdf]

SUPPLEMENTARY INFORMATION

Watching Right and Wrong Nucleotide Insertion Reveals Hidden Polymerase Fidelity Checkpoints

Joonas A. Jamsen[1,*], David D. Shock[1] & Samuel H. Wilson[1,*]

[1]Genome Integrity and Structural Biology Laboratory, National Institute of Environmental Health Sciences, National Institutes of Health, Research Triangle Park, NC 27709, USA.

*Correspondence should be addressed to S.H.W. (email: wilson5@niehs.nih.gov) and J.A.J. (email: joonas.jamsen@nih.gov).

This File Contains:

Supplementary Figures 1-8

Supplementary Tables 1-6

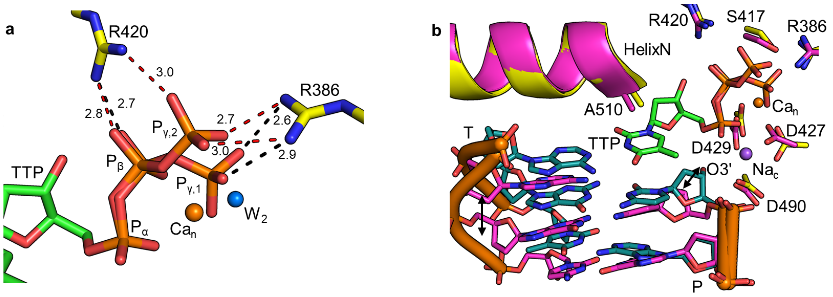

Supplementary Figure 1. Matched Ca^{2+}-ground state ternary complex. a, P_γ dynamics. P_γ can be modeled in two conformations ($P_{\gamma,1}$ and $P_{\gamma,2}$). A water molecule (W_2) replaces the Ca_n interacting oxygen of $P_{\gamma,1}$ and completes the coordination sphere of Ca_n when $P_{\gamma,2}$ interacts with Arg420. Interaction distances (Å) are shown with black dashed lines for $P_{\gamma,1}$ and red dashed lines for $P_{\gamma,2}$. TTP is shown in green stick representation, sidechains in yellow. The orange and blue spheres are Ca^{2+} and water, respectively. **b**, Overlay of the matched Ca^{2+}-GS ternary complex with a pol λ-DNA binary complex (magenta, PDB id 1XSL), the latter lacking an incoming nucleotide. Although template (T) and primer (P) strand shifts are robust upon TTP binding, shifts in α-helix N and triphosphate interacting sidechains are minimal. Shifts in catalytic aspartates accommodate metal binding. Double headed arrows display differences in template strand and O3′ positioning.

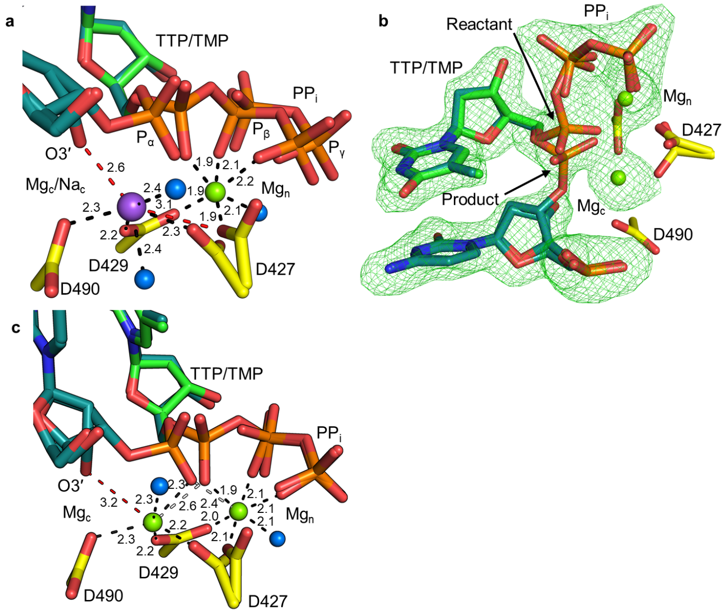

Supplementary Figure 2. Matched Mg^{2+}-reaction state (RS) ternary complex. **a**, Active site metal coordination in the matched (TTP:A_t) Mg^{2+}-RS complex. Coordination distances (2.2-2.6 Å) indicate the catalytic site contains Mg^{2+} and Na^{+}, the latter was modeled in this site. TMP coordination of the atoms in the catalytic metal site (Mg_c/Na_c) is shown with black dashes, alternate coordination of a P_α oxygen of TTP is shown with a red line. Coordination of the nucleotide Mg^{2+} (Mg_n) is shown with black dashes. Green, purple and blue spheres represent Mg^{2+}, Na^{+} and water molecules, respectively. **b**, Shorter (1.5 min) Mg^{2+} soak confirms catalytic site occupancy by Mg^{2+}. Arrows indicate bond broken (substrate) and formed (product). Simulated annealing (F_o-F_c) omit density (green mesh) shown is contoured at 3 σ, carve radius 2.0 Å. **c**, Active site metal coordination in the 1.5 min Mg^{2+} soak. Coordination distances (2.2-2.3 Å) are consistent with Mg_c bound in the catalytic site.

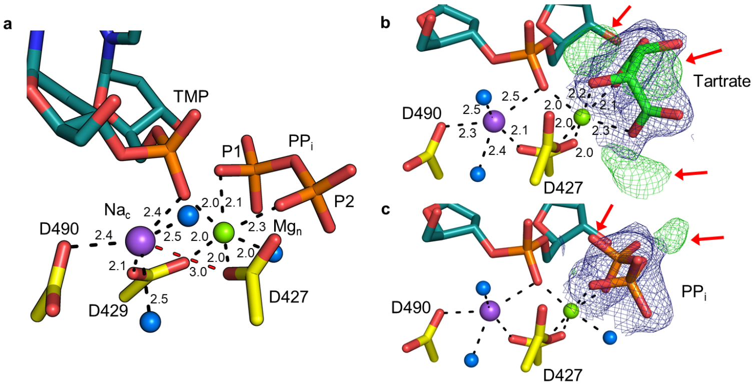

Supplementary Figure 3. Matched Mg^{2+}-product (PS) ternary complex. a, Metal coordination in the Mg^{2+}-product state (PS_{Mg}) of matched (TTP:A_t) insertion. Coordination geometry and distances are consistent with Na_c (2.1-2.5 Å) and Mg_n (2.0-2.2 Å). Mg_c has therefore exchanged for Na_c upon product formation. Sidechains are shown in yellow stick representation and DNA is in cyan. Na^+ is the purple sphere, Mg^{2+} a green sphere and water molecules are in blue. Metal coordination (Å) is shown with black dashes and distance (Å) with red dashes. **b**, Matched (TMP:A_t) Mg^{2+}-product complex modeled with tartate. Tartrate partially accounts for the experimental density; areas of positive difference density in locations of PP_i phosphate oxygens appear upon refinement. Tartrate is shown in green stick representation. **c**, Matched TMP:A_t product complex modeled with PP_i. The view is identical to **b**. A red arrow indicates an oxygen of PP_i partially out of density, while a missing covalently bound atom is indicated by positive difference density above the central oxygen, suggestive of P1 dynamics. In **b**, and **c**, the ($2F_o$-F_c) density for PP_i or tartrate (blue mesh) is contoured at 1.5 σ, carve radius 1.0 Å. The positive F_o-F_c density (green mesh) is contoured at 3 σ, carve radius 3.0 Å.

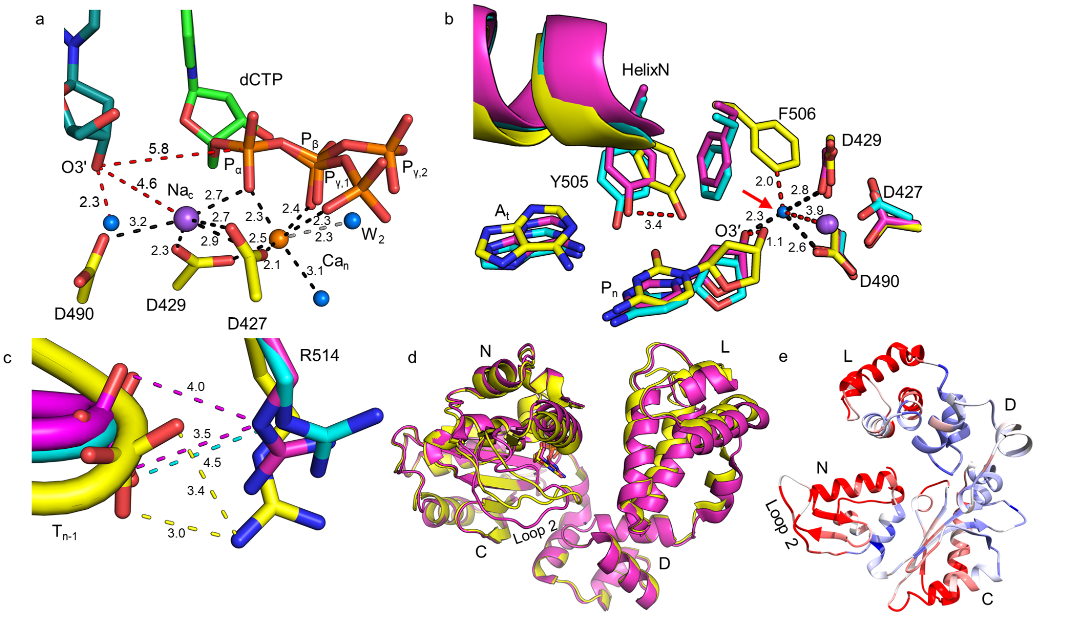

Supplementary Figure 4. Mismatch Ca^{2+}-ground state (GS) ternary complex. a, Active site metal coordination in the mismatch (dCTP:A_t) Ca^{2+}-ground state ternary complex. P_γ is shown in two conformations with an alternate water (W_2). W_2 coordination of Ca_n is shown with a gray dashed line. Metal coordination is shown with black dashes (Å). Red dashes indicate key distances (Å). **b**, Changes in active site residue positions in the mismatch Ca^{2+}-ground state. Shown is an overlay of the matched (TTP, yellow sticks) and mismatched (dCTP, magenta sticks) ground state ternary complexes with a binary complex (PDB id 1XSL, cyan). Shifts in steric gate residues (Y505, F506), primer terminus (P_n), template nucleotide (A_t), and α-helix N accommodate dCTP binding. A water molecule (red arrow) coordinates O3′ and catalytic aspartates. Palm domains (residues 386-494) were aligned to generate the superimposition. **c**, Template strand Arg514 interactions. Shown are the Ca^{2+}-GS matched (yellow) and mismatched (cyan) ternary complexes overlayed with the mismatched Mn^{2+}-PS (magenta) complex. DNA is shown in cartoon representation. Hydrogen bonding (Å) is shown with dashes. **d**, Structural comparison of matched (yellow cartoon) and mismatched (magenta cartoon) Ca^{2+}-GS ternary complexes. Palm domains (residues 386-494) were aligned. Polymerase N (thumb), C (palm), D (fingers) and L (lyase) subdomains are indicated. **e**, Global conformational changes in matched (TTP:A_t) and mismatched (dCTP:A_t) Ca^{2+}-GS complexes. Protein backbones were aligned using the program Matchmaker in Chimera and are colored as a heatmap that corresponds to differences in positions of protein backbone carbon (ΔC_α) atoms from 0 (blue) to 0.5 Å (white) to 1.0+ Å (red). N (thumb), C (palm), D (fingers) and L (lyase) indicate pol λ subdomains.

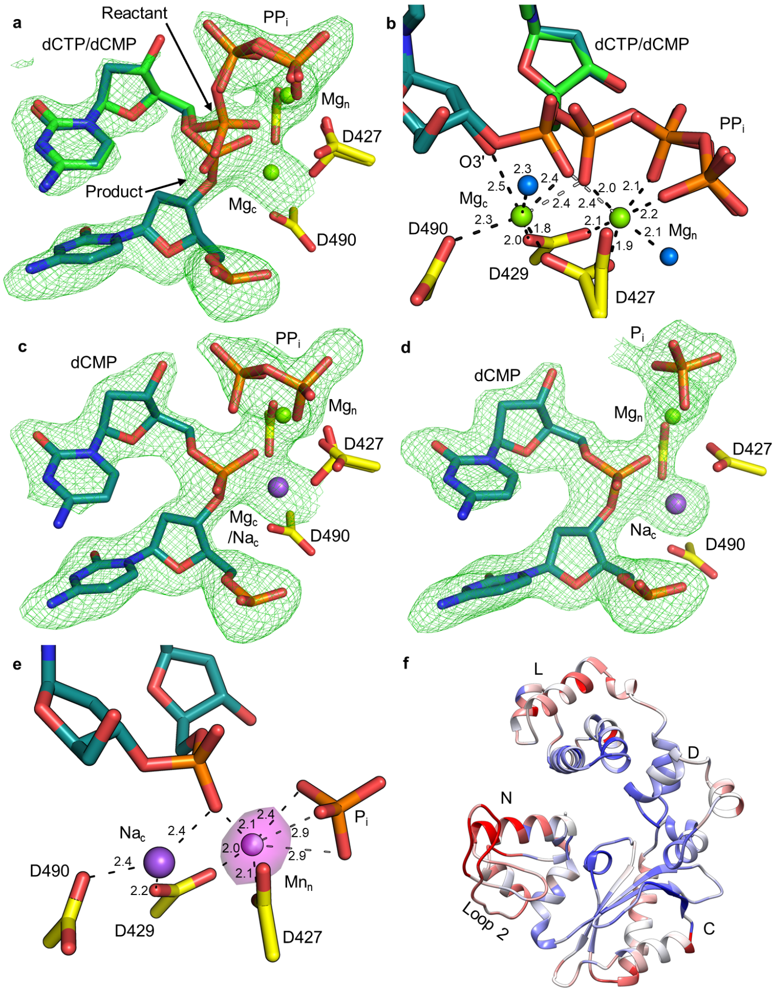

Supplementary Figure 5. Mismatch insertion. a, Mg^{2+}-mismatch reaction state (RS) ternary complex. The arrows indicate bond broken (reactant) and formed (product). dCTP is shown in green stick representation, side chains are in yellow, DNA in cyan. Mg^{2+} are the green spheres. **b**, Active site metal coordination in the mismatch Mg^{2+}-RS. Water molecules are blue spheres. Black and gray dashes indicate metal coordination (Å) with alternate coordination shown as gray dashes. **c**, Mismatch Mg^{2+}-product (PS) complex. The larger purple sphere is Na^{+}. **d**, Extended soak of the mismatch Mg^{2+}-product complex. Reduced density in the PP_i region indicates that P1 (former P_β of TTP) of PP_i has dissociated. **e**, Metal coordination in the extended soak of the Mn^{2+} mismatch product complex (PS^{ES}). Mn^{2+} is the smaller magenta sphere. Black and gray dashes indicate metal coordination (Å). The anomalous map (magenta surface) shown is contoured at 5 σ, carve radius 2.0 Å. **f**, Global conformational changes in matched (TTP:A_t) and mismatched (dCTP:A_t) Mn^{2+}-PS complexes. Protein backbones were aligned using Matchmaker in Chimera and are colored as a heatmap corresponding to differences in positions of protein backbone carbon (ΔC_α) atoms from 0 (blue) to 0.5 Å (white) to 1.0 Å and above (red). N (thumb), C (palm), D (fingers) and L (lyase) indicate pol λ subdomains.

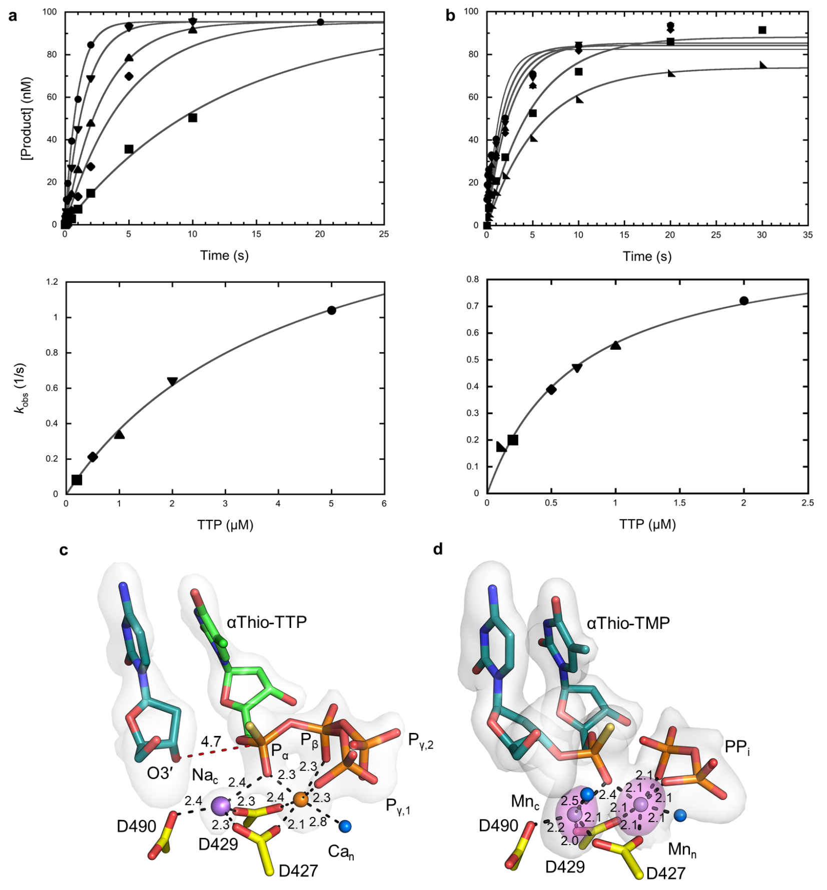

Supplementary Figure 6. Modulating the product metal. a, Single-turnover analysis of TTP insertion opposite A_t in the presence of Mg^{2+}. Time courses are shown in the top panel and secondary plot in the bottom panel. **b**, Single-turnover time courses (top) and secondary plot (bottom) of TTP insertion opposite A_t in the presence of Mn^{2+}. Source data for panels **a** and **b** are provided with this paper. **c**, Ground state (GS) ternary Ca^{2+}-complex with incoming TTPαS. The simulated annealing omit (F_o-F_c) density shown in gray surface representation is contoured at 3.0 σ, carve radius 2.0 Å. Black and red dashed lines show metal coordination or atomic distance of O3′ from P_α, respectively. TTPαS is shown in green stick representation (sulfur atom is in dark yellow), DNA in cyan, sidechains in yellow. Ca^{2+} is the orange sphere, Na^{+} is the larger purple sphere, water is blue. **d**, Product state ternary complex of Mn^{2+}-mediated TTPαS insertion opposite A_t after a 60 min soak. Mn^{2+} is the smaller magenta sphere. The simulated annealing omit (F_o-F_c) density shown (gray surface) is contoured at 3.0 σ, carve radius 2.0 Å.

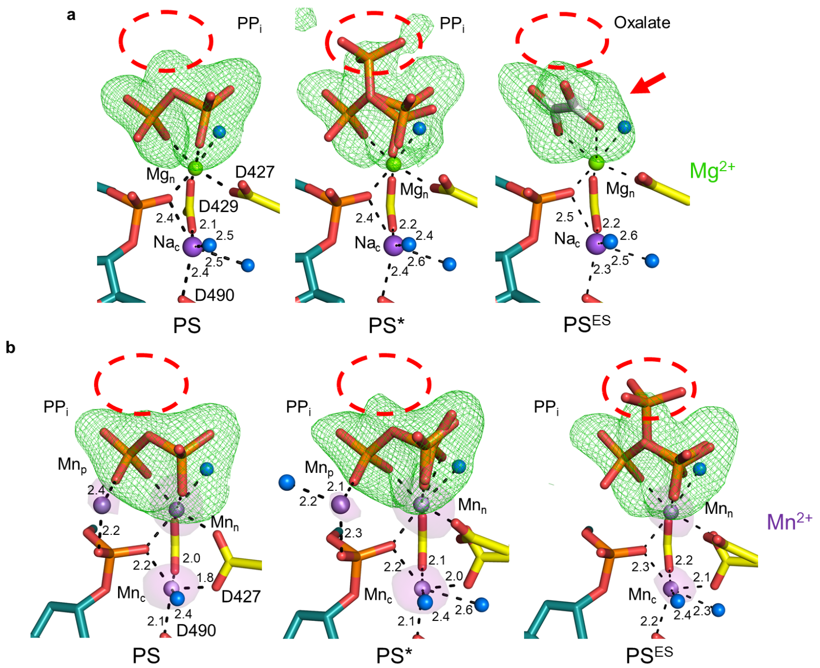

Supplementary Figure 7. Pyrophosphate and product metal release. a, Post-catalytic events in the matched Mg^{2+} reaction. P1 dynamics in the product state (left, PS) and extended soaks (middle and right, PS* and PS^{ES}) is shown as changes in the simulated annealing omit density (green mesh) contoured at 3 σ, carve radius 2 Å. Red ovals indicate density corresponding to either an alternate conformation of P1 or a component of the cryo-solution, tartrate (see Supplementary Fig. 3b-c). Asp427 has rotated into a product conformation, indicating the catalytic site is likely occupied by Na^{+}. PP$_i$ dissociation had occurred after an overnight soak (right, PS^{ES}) as judged from loss of density corresponding to P2 of PP$_i$ (former P$_\gamma$ of TTP). The remaining density was modeled as oxalate, a potential cryo-contaminant. A red arrow indicates loss of density for a P2 phosphate oxygen. Sidechains are in yellow stick representation, DNA in cyan. Mg$_n$ is a green sphere, Na$_c$ is a large purple sphere, water molecules are blue spheres. Metal coordination (Å) is shown with black dashes. **b**, Post-catalytic events in the matched Mn^{2+} reaction. Decreased P1 dynamics is observed in the Mn^{2+}-mediated insertion compared to Mg^{2+}. The product metal is still present in the 60 min soak (middle, PS*), but has completely dissociated after the overnight soak (right, PS^{ES}). Anomalous density is shown as a magenta surface contoured at 5 σ, carve radius 2 Å.

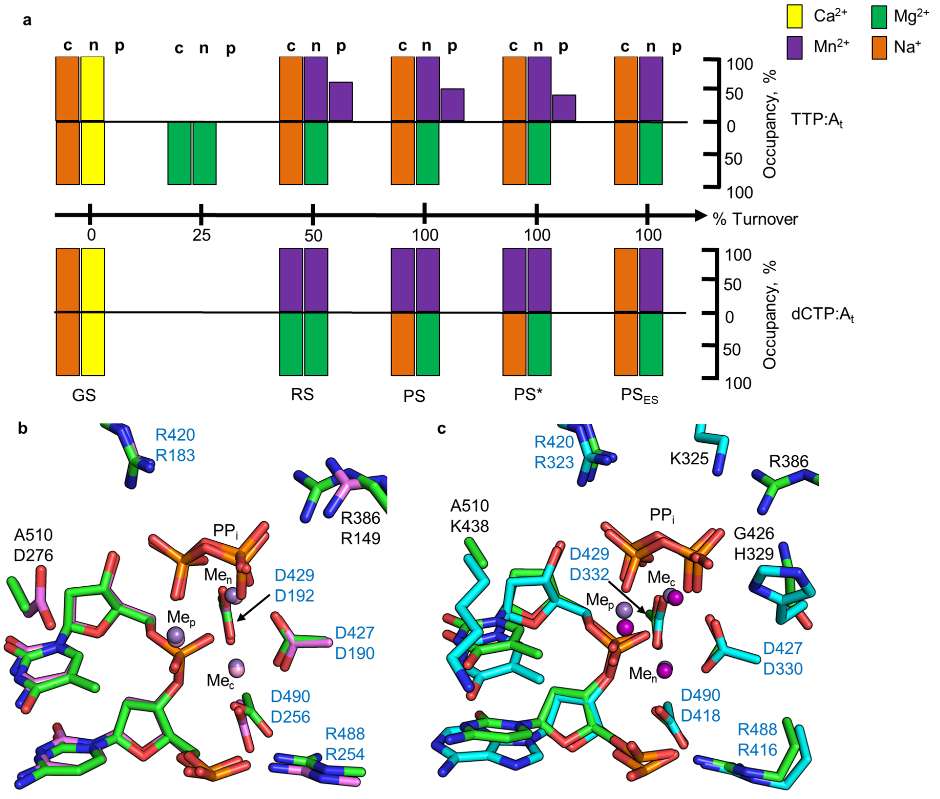

Supplementary Figure 8. Active site metal dynamics. a, Modelling of occupancies of the catalytic (c), nucleotide (n) and product metal (p) binding sites during Mg^{2+}- and Mn^{2+}-mediated matched (TTP:A_t) and mismatched (dCTP:A_t) nucleotide insertion. The central black line indicates degree of nucleotide incorporation (% Turnover). **b**, Comparison of the Mn^{2+}-product ternary complexes of pols λ (20 min TTP Mn^{2+} soak) and β (PDB id 4KLH). The catalytic domains (pol λ residues 386-494; pol β residues 149-260) of each polymerase were aligned to generate the superimposition. **c**, Comparison of the Mn^{2+}-product ternary complexes of pols λ (20 min TTP Mn^{2+} soak) and μ (PDB id 5TYX). The catalytic domains (pol λ residues 386-494; pol μ residues 289-522) were aligned in PyMol. In panels **b** and **c**, DNA and sidechains are shown in green for pol λ, pink for pol β and cyan for pol μ. Mn^{2+} is shown as magenta, pink and purple spheres for pols λ, β and μ, respectively. Residues labeled in blue indicate similar position and likely role in the active site, whereas black labels indicate a different position and predicted function.

Supplementary Table 1. Crystallographic statistics for TTP insertion opposite A_t in the presence of Ca^{2+} or Mg^{2+}.

	TTP:dA Ca^{2+}	TTP:dA Mg^{2+}, 1.5 min	TTP:dA Mg^{2+}, 2 min	TTP:dA Mg^{2+}, 5 min	TTP:dA Mg^{2+}, 60 min	TTP:dA Mg^{2+}, 960 min
**PDB ID**	7M43	7M44	7M45	7M46	7M47	7M48
**Data Collection**						
Space Group	$P2_12_12_1$	$P2_12_12_1$	$P2_12_12_1$	$P2_12_12_1$	$P2_12_12_1$	$P2_12_12_1$
Cell Dimensions						
a, b, c (Å)	55.988	56.042	56.018	56.263	56.049	56.168
	62.653	62.356	62.547	62.808	62.462	62.653
	140.386	140.228	140.010	139.452	140.059	139.912
α, β, γ (°)	90, 90, 90	90, 90, 90	90, 90, 90	90, 90, 90	90, 90, 90	90, 90, 90
Resolution (Å)[1]	50 – 1.98	50 – 1.80	50 – 1.89	50 – 1.92	50 – 1.65	50 – 1.93
	(2.05 – 1.98)	(1.97 – 1.90)	(1.96 – 1.89)	(1.99 – 1.92)	(1.71 – 1.65)	(2.00 – 1.93)
R_{sym} or R_{merge}[1]	7.0 (50.3)	5.7 (83.6)	6.4 (54.5)	5.7 (59.1)	6.8 (51.7)	8.9 (94.5)
$I/\sigma I$[1]	21.0 (2.0)	38.0 (2.1)	34.0 (2.42)	26.9 (2.1)	25.2 (2.7)	22.5 (2.2)
Completeness (%)[1]	99.3 (92.8)	99.8 (100.0)	98.9 (88.5)	99.8 (98.6)	99.3 (91.6)	99.8 (99.9)
Redundancy[1]	6.5 (3.2)	11.5 (6.4)	11.6 (4.8)	5.9 (3.0)	7.1 (5.7)	7.4 (7.2)
No. Unique Refl.[1]	35,360	39,547	40,323	38,634	60,175	38,022
**Refinement**						
RS : PS (%)	100 : 0	70 : 30	40 : 60	0 : 100	0 : 100	0 : 100
A site occ (%)	Na^+:100	Mg^{2+}:100	Na^+:100	Na^+:100	Na^+:100	Na^+:100
B site occ (%)	Ca^{2+}:100	Mg^{2+}:100	Mg^{2+}:100	Mg^{2+}:100	Mg^{2+}:100	Mg^{2+}:100
C site occ (%)	–	–	–	–	–	–
Resolution (Å)	24 – 1.98	22 – 1.90	24 – 1.89	35 – 1.64	35 – 1.65	50 – 1.93
No. Reflections	34,913	38,868	39,210	38,262	59,624	37,872
R_{work} / R_{free}	0.19 / 0.23	0.20 / 0.23	0.21 / 0.24	0.19 / 0.22	0.18 / 0.21	0.18 / 0.22
No. atoms						
Protein / DNA	2390 / 438	2367 / 465	2437 / 507	2458 / 465	2501 / 446	2433 / 446
dNTP / PP_i / Metal	38 / – / 6	29 / 9 / 5	29 / 9 / 5	– / 9 / 4	– / 9 / 5	– / – / 5
Water / Ligands	248 / 66	241 / 66	250 / 57	251 / 30	294 / 40	255 / 28
B-factors						
Protein / DNA	43.8 / 32.7	44.5 / 32.8	43.8 / 32.0	44.7 / 34.6	40.5 / 29.9	44.1 / 31.6
Me_A / Lig_A[2]	39.2 / 37.1	30.5 / 27.0	30.5 / 28.3	31.6 / 31.9	24.1 / 23.8	29.6 / 27.0
Me_B / Lig_B[2]	36.3 / 34.7	25.4 / 27.7	25.8 / 31.4	28.0 / 32.6	20.2 / 26.4	27.1 / 29.3
Me_C / Lig_C[2]	–	–	–	–	–	–
Water / Ligands[3]	39.5 / 36.5	41.2 / 39.1	41.2 / 36.5	42.0 / 49.4	40.4 / 47.0	41.8 / 52.3
Wilson B	30.0	31.0	30.4	33.1	25.8	29.8
R.M.S Deviations						
Bond Lengths (Å)	0.009	0.011	0.007	0.007	0.010	0.007
Bond Angles (°)	0.927	1.260	1.061	1.040	1.207	0.868

[1]Data in the highest resolution shell is shown in the parenthesis.
[2]B-factors for the catalytic metal (Me_A), nucleotide metal (Me_B), product metal (Me_C), and valence weighted environmental average of surrounding ligands (Lig_A, Lig_B and Lig_C).
[3]Overall B-factor for ligands/solutes.

Supplementary Table 2. Crystallographic statistics for dCTP insertion opposite A_t in the presence of Ca^{2+} or Mg^{2+}.

	dCTP:dA Ca^{2+}	dCTP:dA Mg^{2+}, 120 min	dCTP:dA Mg^{2+}, 300 min	dCTP:dA Mg^{2+}, 960 min
**PDB ID**	7M49	7M4A	7M4B	7M4C
**Data Collection**				
Space Group	$P2_12_12_1$	$P2_12_12_1$	$P2_12_12_1$	$P2_12_12_1$
Cell Dimensions				
a, b, c (Å)	55.750	55.825	55.802	55.997
	59.917	62.088	62.395	62.235
	141.302	142.096	142.482	141.114
α, β, γ (°)	90, 90, 90	90, 90, 90	90, 90, 90	90, 90, 90
Resolution (Å)[1]	50 – 1.82	50 – 1.90	50 – 1.92	50 – 1.88
	(1.88 – 1.82)	(1.97 – 1.90)	(2.02 – 1.95)	(1.95 – 1.88)
R_{sym} or R_{merge}[1]	9.2 (85.9)	8.1 (96.0)	7.1 (78.5)	5.0 (82.6)
$I/\sigma I$[1]	18.3 (2.2)	16.8 (2.0)	21.3 (2.1)	32.6 (2.2)
Completeness (%)[1]	99.7 (99.8)	99.4 (96.0)	99.8 (100.0)	99.8 (99.3)
Redundancy[1]	6.7 (6.2)	6.0 (5.1)	6.0 (5.5)	5.8 (5.5)
No. Unique Refl.[1]	43,513	39,649	38,401	41,021
**Refinement**				
RS : PS (%)	100 : 0	60 : 40	0 : 100	0 : 100
A site occ (%)	Na^{+}:100	Mg^{2+}:100	Na^{2+}:100	Na^{+}:100
B site occ (%)	Ca^{2+}:100	Mg^{2+}:100	Mg^{2+}:100	Mg^{2+}:100
C site occ (%)	–	–	–	–
Resolution (Å)	40 – 1.82	50 – 1.90	50 – 1.92	40 – 1.88
No. Reflections	43,319	39,326	36,477	40,846
R_{work} / R_{free}	0.21 / 0.25	0.21 / 0.23	0.21 / 0.24	0.21 / 0.24
No. atoms				
Protein / DNA	2353 / 466	2305 / 504	2309 / 445	2325 / 527
dNTP / PP_i / Metal	36 / 0 / 3	28 / 9 / 4	0 / 9 / 4	0 / 5 / 4
Water / Ligands	202 / 55	127 / 45	133 / 14	146 / 22
B-factors				
Protein / DNA	52.9 / 44.3	59.3 / 52.3	56.8 / 53.2	56.8 / 50.7
Me_A / Lig_A[2]	59.1 / 56.1	48.8 / 49.8	44.1 / 52.1	46.0 / 54.1
Me_B / Lig_B[2]	63.7 / 69.2	57.6 / 55.8	44.5 / 49.9	70.3 / 72.9
Me_C / Lig_C[2]	–	–	–	–
Water / Ligands[3]	54.9 / 74.2	56.2 / 62.9	50.5 / 54.5	52.2 / 64.1
Wilson B	35.0	42.9	41.8	40.3
R.M.S Deviations				
Bond Lengths (Å)	0.012	0.012	0.013	0.007
Bond Angles (°)	1.141	1.323	1.295	0.945

[1]Data in the highest resolution shell is shown in the parenthesis.
[2]B-factors for the catalytic metal (Me_A), nucleotide metal (Me_B), product metal (Me_C), and valence weighted environmental average of surrounding ligands (Lig_A, Lig_B and Lig_C).
[3]Overall B-factor for ligands/solutes.

Supplementary Table 3. Crystallographic statistics for dCTP insertion opposite A_t in the presence of Mn^{2+}.

	dCTP:dA Mn^{2+}, 225 min	dCTP:dA Mn^{2+}, 420 min	dCTP:dA Mn^{2+}, 960 min
**PDB ID**	7M4D	7M4E	7M4F
**Data Collection**			
Space Group	$P2_12_12_1$	$P2_12_12_1$	$P2_12_12_1$
Cell Dimensions			
a, b, c (Å)	55.858	55.750	55.975
	62.546	62.317	63.660
	142.082	142.177	139.970
α, β, γ (°)	90, 90, 90	90, 90, 90	90, 90, 90
Resolution (Å)[1]	50 – 1.93	50 – 2.00	42 – 2.40
	(1.99 – 1.92)	(2.08 – 2.01)	(2.46 – 2.38)
R_{sym} or R_{merge}[1]	8.5 (93.8)	7.0 (82.3)	6.5 (62.0)
$I/\sigma I$[1]	21.4 (1.9)	18.8 (1.7)	12.6 (1.6)
Completeness (%)[1]	100.0 (99.7)	99.1 (93.8)	94.7 (84.1)
Redundancy[1]	5.8 (4.9)	4.8 (4.8)	2.4 (1.7)
No. Unique Refl.[1]	38,336	33,936	20,027
**Refinement**			
RS : PS (%)	40 : 60	0 : 100	0 : 100
A site occ (%)	Mn^{2+}:100	Mn^{2+}:80	Na^{+}:100
B site occ (%)	Mn^{2+}:100	Mn^{2+}:90	Mn^{2+}:90
C site occ (%)	–	–	–
Resolution (Å)	50 – 1.93	50 – 2.00	50 – 2.40
No. Reflections	38,236	33,546	18,915
R_{work} / R_{free}	0.19 / 0.23	0.22 / 0.25	0.23 / 0.26
No. atoms			
Protein / DNA	2458 / 546	2303 / 527	2313 / 445
dNTP / PP_i / Metal	28 / 9 / 5	0 / 9 / 6	0 / 5 / 5
Water / Ligands	242 / 50	82 / 26	68 / 24
B-factors			
Protein / DNA	48.2 / 40.3	60.2 / 56.7	54.1 / 51.1
Me_A / Lig_A[2]	33.8 / 30.6	56.6 / 53.2	33.4 / 42.0
Me_B / Lig_B[2]	31.0 / 29.9	50.1 / 54.1	30.6 / 51.2
Me_C / Lig_C[2]	–	–	–
Water / Ligands[3]	45.2 / 33.9	56.1 / 52.7	40.4 / 52.5
Wilson B	33.0	46.2	44.3
R.M.S Deviations			
Bond Lengths (Å)	0.008	0.003	0.003
Bond Angles (°)	0.904	0.649	0.618

[1]Data in the highest resolution shell is shown in the parenthesis.
[2]B-factors for the catalytic metal (Me_A), nucleotide metal (Me_B), product metal (Me_C), and valence weighted environmental average of surrounding ligands (Lig_A, Lig_B and Lig_C).
[3]Overall B-factor for ligands/solutes.

Supplementary Table 4. Crystallographic statistics for TTP and TTPαS insertion opposite A_t with Ca^{2+} or Mn^{2+}.

	TTP:dA Mn^{2+}, 5 min	TTP:dA Mn^{2+}, 20 min	TTP:dA Mn^{2+}, 60 min	TTP:dA Mn^{2+}, 960 min	TTPαS:dA Ca^{2+}	TTPαS:dA Mn^{2+}, 60 min
	7M4G	7M4H	7M4I	7M4J	7M4K	7M4L
**Data Collection**						
Space Group	$P2_12_12_1$	$P2_12_12_1$	$P2_12_12_1$	$P2_12_12_1$	$P2_12_12_1$	$P2_12_12_1$
Cell Dimensions						
a, b, c (Å)	56.146	56.050	55.981	56.021	56.001	55.970
	62.771	62.727	62.634	62.641	62.507	62.573
	140.568	140.405	140.334	140.120	140.191	140.130
α, β, γ (°)	90, 90, 90	90, 90, 90	90, 90, 90	90, 90, 90	90, 90, 90	90, 90, 90
Resolution (Å)[1]	50 – 1.60	50 – 1.87	50 – 1.66	50 – 1.95	50 – 1.72	50 – 1.70
	(1.66 – 1.60)	(1.93 – 1.87)	(1.72 – 1.66)	(2.02 – 1.95)	(1.78 – 1.72)	(1.76 – 1.70)
R_{sym} or R_{merge}[1]	5.3 (77.4)	7.8 (89.9)	7.5 (54.2)	9.5 (96.7)	10.6 (43.7)	7.0 (71.5)
$I/\sigma I$[1]	39.7 (2.6)	23.0 (2.9)	22.8 (1.9)	25.0 (2.9)	16.0 (3.0)	22.8 (1.9)
Completeness (%)[1]	99.5 (93.8)	99.9 (100.0)	97.4 (82.6)	100.0 (100.0)	98.9 (90.4)	97.4 (82.6)
Redundancy[1]	10.4 (9.3)	7.0 (5.9)	6.4 (5.0)	8.8 (8.7)	5.7 (4.6)	6.4 (5.0)
No. Unique Refl.[1]	66,477	41,883	55,046	36,809	53,151	55,046
**Refinement**						
RS : PS (%)	50 : 50	0 : 100	0 : 100	0 : 100	100 : 0	0 : 100
A site occ (%)	Mn^{2+}:100	Mn^{2+}:100	Mn^{2+}:100	Mn^{2+}:100	Na^{+}:100	Mn^{2+}:100
B site occ (%)	Mn^{2+}:100	Mn^{2+}:100	Mn^{2+}:100	Mn^{2+}:100	Ca^{2+}:100	Mn^{2+}:100
C site occ (%)	Mn^{2+}:60	Mn^{2+}:50	Mn^{2+}:40	–	–	–
Resolution (Å)	35 – 1.60	35 – 1.87	30 – 1.66	50 – 1.95	50 – 1.72	50 – 1.70
No. Reflections	65,991	41,765	53,486	36,725	52,437	53,481
R_{work} / R_{free}	0.19 / 0.22	0.19 / 0.22	0.18 / 0.20	0.18 / 0.21	0.20 / 0.23	0.19 / 0.22
No. atoms						
Protein / DNA	2486 / 507	2384 / 488	2502 / 507	2463 / 507	2361 / 426	2470 / 468
dNTP / PP_i / Metal	29 / 9 / 7	0 / 9 / 7	0 / 9 / 7	0 / 9 / 6	29 / 0 / 5	0 / 9 / 5
Water / Ligands	276 / 62	171 / 36	265 / 39	244 / 34	201 / 56	248 / 53
B-factors						
Protein / DNA	47.3 / 34.3	52.1 / 40.1	44.9 / 32.2	43.8 / 31.3	50.9 / 39.4	47.8 / 35.3
Me_A / Lig_A[2]	28.5 / 31.2	37.8 / 39.4	28.7 / 29.1	37.7 / 29.1	48.7 / 49.8	45.8 / 34.7
Me_B / Lig_B[2]	26.6 / 29.5	34.5 / 42.8	25.2 / 31.1	23.5 / 30.1	45.6 / 61.3	31.3 / 37.2
Me_C / Lig_C[2]	40.1 / 34.0	47.2 / 46.9	31.4 / 34.9	–	–	–
Water / Ligands[3]	50.4 / 46.2	47.7 / 54.8	43.4 / 50.1	42.0 / 40.3	47.7 / 55.9	42.8 / 39.6
Wilson B	35.2	37.8	28.6	29.9	32.6	31.6
R.M.S Deviations						
Bond Lengths (Å)	0.011	0.008	0.011	0.011	0.010	0.015
Bond Angles (°)	1.217	1.158	1.199	1.245	1.174	1.344

[1]Data in the highest resolution shell is shown in the parenthesis.

[2]B-factors for the catalytic metal (Me_A), nucleotide metal (Me_B), product metal (Me_C), and valence weighted environmental average of surrounding ligands (Lig_A, Lig_B and Lig_C).

[3]Overall B-factor for ligands/solutes.

Supplementary Table 5. Kinetics of matched gap-filling insertion by pol λ[1].

Me^{2+}	k_{pol}	$K_{d,TTP}$	k_{pol}/K_d
	s^{-1}	μM	$s^{-1}\mu M^{-1}$
Mg[a]	n.d.[b]	n.d.[b]	0.45 ± 0.03[c]
Mn	0.96 ± 0.08	0.7 ± 0.1	1.4 ± 0.2

[1]Source data are provided with this paper.

[a]Assays were performed at 10 mM Mg^{2+} or 1 mM Mn^{2+}

[b]n.d., not estimated due to observed double exponential time courses at high [TTP]

[c]Catalytic efficiency was determined by fitting time courses at sub-saturating [TTP] to the equation $k_{obs} = (k_{cat}/K_d)[TTP]/(1 + [TTP]/K_d)$

Supplementary Table 6. Sequences of oligonucleotides used in this study.

Experiment	Designation	Sequence (5'-3'), template base underlined
Kinetics	Template	CATTGACGCTCGGC A GTACTGACTGCATATCACCG
Kinetics	Upstream	[6-FAM]CGGTGATATGCAGTCAGTAC
Kinetics	Downstream	pGCCGAGCGTCAATG
Crystallography	Template	CGGC A GTACTG
Crystallography	Upstream	CAGTAC
Crystallography	Downstream	pGCCG
